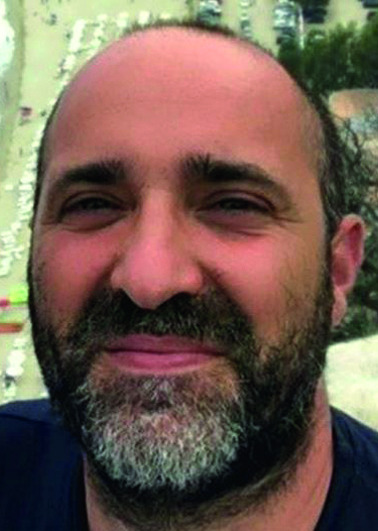# Advancing CO_2_ Conversion with Cu‐LDHs: A Review of Computational and Experimental Studies

**DOI:** 10.1002/tcr.202500014

**Published:** 2025-04-14

**Authors:** Fabio Loprete, Eleonora Tosi Brandi, Francesco Calcagno, Jacopo De Maron, Andrea Fasolini, Riccardo Tarroni, Francesco Basile, Ivan Rivalta

**Affiliations:** ^1^ Dipartimento di Chimica Industriale “Toso Montanari” Alma Mater Studiorum – Università di Bologna Via Piero Gobetti 85 40129 Bologna Italy; ^2^ Center for Chemical Catalysis – C3, Alma Mater Studiorum – Università di Bologna Via Piero Gobetti 85 40129 Bologna Italy; ^3^ Interdepartmental Center for Industrial Research, Renewable Sources, Environment, Sea, Energy (CIRI-FRAME), Alma Mater-Studiorum Università di Bologna Via Piero Gobetti 85 40129 Bologna Italy

**Keywords:** Layered Double Hydroxide, CO_2_ reduction, Photocatalysis, Electrocatalysis, Copper-based Systems

## Abstract

Layered Double Hydroxides (LDHs) are versatile materials with tuneable properties. They show promising electro‐ and photo‐catalytic activity in the activation and conversion of CO_2_. Their unique properties make LDHs pivotal materials in emerging sustainable strategies for mitigating the effect of CO_2_ emissions. However, the intricate structure‐property relationship inherent to LDHs challenges their rational design. In this review, we provide a comprehensive overview of both experimental and computational studies about LDHs for photo‐ and electro‐catalytic conversion of CO_2_, mainly focusing on Cu‐based systems due to their superior performance in producing C_2_ products. We present a background framework, describing the essentials computational and experimental tools, designed to support both experimentalists and theoreticians in the development of tailored LDH materials for efficient and sustainable CO_2_ valorisation. Finally, we discuss future potential advancements, emphasizing the importance of new synergistic experimental‐computational studies.

## Graphical abstract

The combination of computational and experimental approaches provides fundamental insights into the electro‐ and photocatalytic conversion of CO_2_ on complex Layered Double Hydroxide‐based catalysts, enabling to advance the understanding of these critical processes

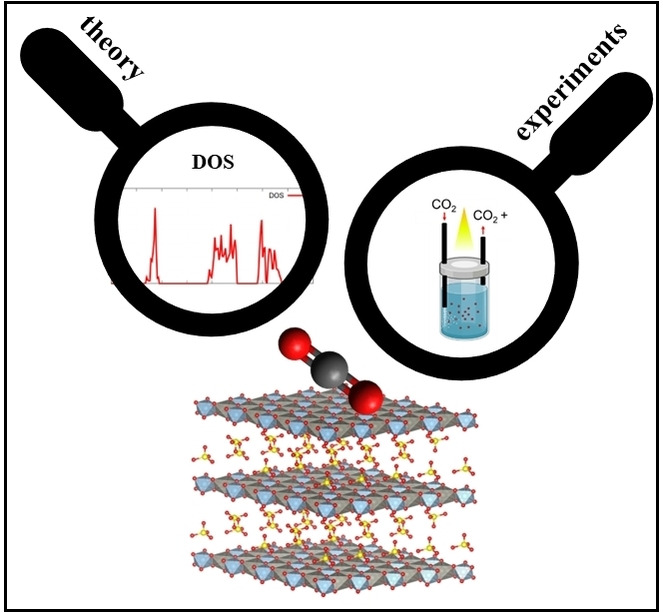

## 1 Introduction

The sustainable conversion of carbon dioxide (CO_2_) into value‐added compounds is an attractive, but extremely challenging research area.[^1^, ^2^] In fact, CO_2_ emissions are largely responsible for climate change, and the conversion of part of CO_2_ would mitigate its environmental impact. However, the thermodynamic stability of CO_2_ challenges the development of effective strategies. In fact, this is the most stable product of the aerobic combustion of hydrocarbons, and its carbon‐oxygen (C=O) double bond is much stronger (750 kJ mol^−1^) than the single carbon‐carbon (C−C, 336 kJ mol^−1^) and carbon‐hydrogen (C−H, 430 kJ mol^−1^) bonds in organic molecules.^[3]^

Over the past few decades, extensive research efforts have been done to develop homogeneous[^4^, ^5^, ^6^] and heterogeneous[^7^, ^8^, ^9^] catalysts able to facilitate at different conditions CO_2_ activation and its selective reduction reaction (CO_2_RR) into valuable compounds, i. e. building block chemicals (e. g. CO, CH_4_, CH_3_OH, CH_3_CH_2_OH, C_2_H_4_)^[10]^ and liquid fuels.^[11]^

**Figure 1:**
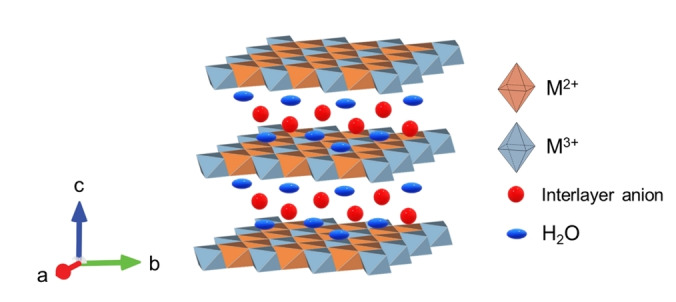
Schematic representation of a LDH structure.

In particular, thermal,^[12]^ electro‐,[^5^, ^13^] photo‐,[^14^, ^15^] and photoelectro‐catalysts^[2]^ have been synthesized, developed and studied. Remarkably, heterogeneous systems have shown better performance and cutting‐edge solutions. For instance, Cu‐based materials and Cu‐based metal oxide supported catalysts perform well in thermocatalysis,[^16^, ^17^] but due to the sluggish kinetics of CO_2_ reduction reactions harsh reaction conditions, i. e. high temperature (≥500 K) and pressure (≥10 bar), are usually required.^[18]^ Instead, electro‐, photo‐catalysis and synergic photo‐electrocatalysis are valuable alternatives since they can achieve the activation and conversion of CO_2_ at room temperature and atmospheric pressure. Still, this requires the development of efficient catalysts featuring appropriate interaction with CO_2_ for its activation even at room temperature, and specific electronic properties to efficiently drive the energy coming from light and/or electricity towards the CO_2_ conversion process.[^3^, ^19^] This is particularly true for diluted steams that are of the greatest interest. In this context, the most impactful and challenging approach would be the direct air capture and conversion (DACC) for which the catalyst‐CO_2_ interaction is crucial. Among heterogeneous systems active in electro‐ and photo‐catalysis, Layered Double Hydroxides (LDHs) have emerged as attractive systems for CO_2_ activation and conversion.[^20^, ^21^, ^22^] LDHs, often referred to as *anionic clays*, are renowned for their cost‐effectiveness, facile synthesis, low toxicity, and remarkable stability.^[23]^ These interesting materials are composed of cationic metal 2D hydroxide layers (M^2+^/M^3+^), interlayer anions (A^−^ or A^2−^), being CO_3_^2−^ the most favoured for charge compensation, and crystallization water molecules, as shown in Figure 1. Their preferential interaction with carbonates results in a complex equilibrium with CO_2_.[^24^, ^25^] Therefore, this peculiar characteristic enlightens the opportunities of deepening the knowledge of these materials in boosting the various steps of the CO_2_ activation and conversion. The large variability in the LDH chemical composition has a positive impact on their current and potential application in catalysis. In fact, electronic properties (e. g. band gap, work function), surface properties (e. g. redox sites, acid‐base sites), and reactivity can be largely tuned by varying the chemical composition and selecting proper synthesis/modification method.[^26^, ^27^, ^28^] For these reasons, LDHs have been considered for materials engineering that enhances the catalytic activity and the selectivity towards desired products. As shown in Table 1, several studies reported the adsorption and, thus, photo‐ and electro‐reduction of CO_2_ by means of LDHs lead to both C_1_ and C_2_ products.[^29^, ^30^, ^31^, ^32^, ^33^, ^34^, ^35^, ^36^, ^37^, ^38^, ^39^, ^40^, ^41^, ^42^, ^43^]

**Table 1:** CO_2_ reduction main products obtained by various LDH‐based photocatalysts and electrocatalysts.[^29^, ^30^, ^31^, ^32^, ^33^, ^34^, ^35^, ^36^, ^37^, ^38^, ^39^, ^40^, ^41^, ^42^, ^43^]

CO_2_ reduction product	Photocatalyst	Electrocatalyst
CO	ZnGa‐LDH,^[29]^ ZnAl‐LDH,^[29]^ CoAl‐LDH,^[42]^ NiAl‐LDH,^[39]^ MgIn‐LDH,^[30]^ MgGa‐LDH,^[30]^ NiGa‐LDH^[30]^	ZnAl‐LDH,^[31]^ ZnAlCe‐LDH,^[32]^ ZnCr‐LDH,^[33]^ CuAl‐LDH^[36]^
CH_4_	ZnTi‐LDH,^[29]^ CoAl‐LDH,^[43]^ NiCoFe‐LDH^[41]^	n/a
CH_3_OH	ZnCuGa‐LDH^[27]^	CuNiAl‐LDH,^[35]^ CuMgAl‐LDH,^[35]^ CuAl‐LDH^[35]^
		
HCOO^−^	n/a	CuAl‐LDH^[36]^, CuNP@MgAl‐LDH^[40]^
CH3COO^−^	n/a	Cu_2_O−Cu@CuMgAl‐LDH^[37]^
C_2_H_4_	n/a	MgAl‐LDH/Cu electrode,^[34]^ CuO@MgAl‐LDH^[38]^

Considering the well‐established ability of Cu‐based materials to facilitate CO_2_ reduction and C−C coupling,[^44^, ^45^] this metal has been considered for LDH‐based materials, achieving highly‐valuable C_2_ products such as C_2_H_4_[^34^, ^38^] and CH_3_COO^−[37]^ (Table 1). This has increased the interest in developing new synthetic methods for Cu‐based LDHs, and in promoting the experimental and computational characterizations of these materials,[^25^, ^40^, ^46^, ^47^, ^48^] which are the focus of this review.

Fundamental insights into the intricate structure‐property relationship of LDHs and LDH‐based catalysts can be given by computational studies, especially when in conjunction with experiments. In fact, the computational modeling of LDH compounds discloses important information about structural, electronic, and reactive properties otherwise difficult to obtain with experiments only.[^49^, ^50^, ^51^] For example, computations allow for comparing several potential active sites and elucidate the effects of surface modifications – such as doping, defect engineering, and surface decoration‐ paving the way for critical insights into experimental observations and rational design of new more efficient systems.[^48^, ^50^, ^52^, ^53^] Prompted by this, we reviewed the relevant advancements in experimental and computational investigations of LDHs for photo‐ and electro‐catalytic processing of CO_2_, with a particular focus on Cu‐based systems. To the best of our knowledge, various reviews have been already published about CO_2_ conversion using LDH‐based materials,[^21^, ^22^, ^26^, ^54^] but never focusing on the role of Cu and the synergy between experimental and computational approaches.

The present work is structured as follows: **Section** **2** discusses the general structure and catalytic properties of LDH systems. **Section** **3** focuses on both experimental and computational methodologies for LDHs characterization and reactivity studies, assessing the toolbox needed by the reader to better appreciate the following discussion and be introduced to state‐of‐the‐art methods used in the field. **Section** **4** and **Section** **5** review experimental and computational studies, respectively, dealing with CO_2_ activation using LDHs. Finally, future directions and outlook of the field are outlined in **Section** **6**, showing a roadmap for advancing the design of LDH‐based systems for sustainable CO_2_ conversion.

## 2 LDHs: From Structure to Catalysis

LDHs are often reported as *hydrotalcite‐like compounds* since they are polytypes and isomorphs of the corresponding Mg−Al based mineral (i. e. Mg_6_Al_2_CO_3_(OH)_16_ ⋅ 4H_2_O). They define a large family of inorganic materials with the general formula [M^2+^_1‐x_M’^3+^_x_(OH)_2_]^x+^(A_x/n_^n^ ⋅ mH_2_O), which suggests the possibility to synthetize a wide set of LDH compounds..^[55]^ For simplicity, we will use the abbreviated form MM’‐A‐LDH in the text, where A may be omitted if not relevant to the discussion hereafter. Among their variability, there are some limiting factors such as the nature of M^2+^ and M’^3+^ and the divalent/trivalent metals ratio (R).^[56]^ Indeed, only cations possessing a radius close to that of Mg^2+^ (0.50 ‐ 0.80 Å) can generally fill in LDH's metal hydroxide layer, namely Ni^2+^, Zn^2+^, Fe^2+^, Co^2+^, Cu^2+^, Al^3+^, Fe^3+^, Cr^3+^, Ga^3+^, Mn^3+^, In^3+[57]^ as well as noble metals (Pt^2+^ Rh^3+^, Ir^3+^, Ru^3+^) .^[58]^ On the other hand, M^+^ and M^4+^ cations as well as large post transition metals such as La^3+^ have been claimed to be potentially inserted in the LDH structure.[^59^, ^60^, ^61^] Furthermore, experimental evidences demonstrated that it is possible to obtain high‐purity LDH only for M^2+^/M’^3+^ ratio (R) between 2 and 6, otherwise single hydroxides or different structures are usually formed.^[55]^ The lower limit of R value depends on the electrostatic repulsion between neighbouring trivalent metal cations and charge‐balancing anionic species in the interlayer. On the other hand, the upper limit corresponds to the condition in which the anions are too little and too distant to prevent the collapse of the interlayer domains.

The hydroxide layers can show different kind of relative disposition, giving rise to different symmetry: rhombohedral or hexagonal.^[62]^ The first occurs more often in natural minerals because it features a lower energy barrier than the second, which in turn is more likely to occur in high‐temperature synthetic LDH compounds.^[62]^

In the interlayer region, LDHs can host a huge variety of charge‐balancing species. Besides crystallization water molecules, other neutral or charged species (e. g. Cl^−^, OH^−^, NO_3_^−^, CO_3_^2−^, SO_4_^2−^, PO_4_^3−^) are present.^[63]^ For instance, LDHs show an intrinsic affinity with carbonates due to their ability to form strong hydrogen bonds that keep the hydroxyl layers very close.^[63]^ In general, the number, the size, the charge, the orientation of interlamellar species and the strength of the bonds between anions and hydroxyl groups has a major impact on the thickness of the interlayer.^[62]^

**Figure 2:**
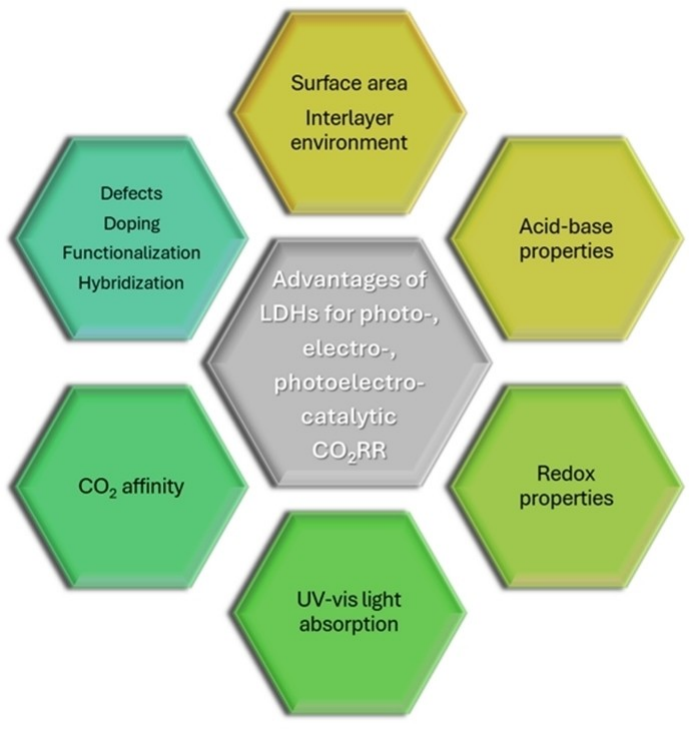
LDHs tuneable features for the design of photo‐, electro‐ and photoelectro‐catalysts.

Different methods of preparation have been found to be suitable to obtain LDHs, namely coprecipitation (i. e. increasing pH method, coprecipitation either at low or at high supersaturation),[^55^, ^64^, ^65^, ^66^] hydrothermal synthesis,[^67^, ^68^] sol‐gel method,[^69^, ^70^] urea method,[^71^, ^72^, ^73^] mechanochemistry,^[74]^ microwave and sonication,[^75^, ^76^] exchange methods (i. e. ion exchange, rehydration or reconstruction, liquid assisted grinding),[^77^, ^78^] electrosynthesis,[^79^, ^80^, ^81^, ^82^, ^83^] and exfoliation.[^84^, ^85^] Some of these techniques can be exploited for both the synthesis and the post‐synthesis modification of LDHs,^[86]^ but the preparation method mostly optimized for LDHs’ synthesis is the coprecipitation,[^55^, ^87^, ^88^, ^89^, ^90^] which provides crystalline products through a reproducible and scalable set‐up. The compositional and structural tunability of LDHs provide numerous opportunities for engineering and design. This versatility allows for tailored properties, enabling LDHs to find application across diverse fields^[91]^ as anion exchangers,^[23]^ flame retardants,^[55]^ electrochemical sensors,^[92]^ drug delivery,^[87]^ polymers filling,^[93]^ and catalysis,[^94^, ^95^, ^96^, ^97^, ^98^, ^99^, ^100^, ^101^, ^102^, ^103^] including both electro‐[^40^, ^104^, ^105^, ^106^, ^107^] and photo‐catalysis[^28^, ^30^, ^108^, ^109^, ^110^, ^111^, ^112^, ^113^, ^114^, ^115^, ^116^] (Table 1). LDHs and their thermal decomposition products (i. e. high‐surface area mixed oxides) are well studied and widely applied for basic catalysis such as polymerization of alkene oxides,^[117]^ alkylation,[^118^, ^119^] aldol condensation,^[120]^ reforming of hydrocarbons,[^94^, ^121^, ^122^] hydrogenation reactions (production of methane, methanol, higher alcohols, paraffins and olefines from syngas, hydrogenation of nitrobenzene),[^123^, ^124^, ^125^] oxidation reactions,[^126^, ^127^, ^128^] and support for Ziegler‐Natta catalysts.^[55]^ In recent years, LDHs have also gained prominence for their photocatalytic and electrocatalytic properties (Figure 2), particularly in the activation and conversion of small molecules such as H_2_O,[^129^, ^130^] CO_2,_[^22^, ^131^] and more recently also N_2_.[^132^, ^133^, ^134^] Their semiconducting properties, crucial for both electro‐ and photo‐catalysis, can be tailored by varying the metal composition of the hydroxide layer, with bandgap energies ranging from 1.34 eV for Ni_2_Fe−Cl‐LDH to 4.63 eV for MgAl−Cl‐LDH.^[27]^ Engineering strategies, such as the creation of oxygen vacancies, have proven effective in enhancing the photocatalytic performance.^[21]^ Oxygen vacancies help preventing charge carrier recombination, creating coordinatively unsaturated sites that facilitate reactant adsorption and activation. Elemental doping offers another route to improving LDH performance, influencing the band structure and promoting charge separation and transfer.^[135]^ In electrocatalysis, the electrical conductivity and electron‐transfer kinetics of LDHs can be enhanced by combining them with conductive materials, such as carbon nanotubes, N‐doped graphene, and reduced graphene oxide (GO).[^22^, ^136^] Additionally, exfoliation techniques have been employed to produce 2D LDH nanosheets with high surface area (and large number of active sites) and quantum confinement effects in one direction.^[137]^ These properties have shown great potential for adsorption as well as photo‐ and electro‐catalytic processes.[^49^, ^138^] Considering the basicity of the hydroxide layers and the characteristics of the interlayer region, LDHs can strongly interact with CO_2_, making them promising catalysts for its conversion.[^26^, ^28^, ^111^, ^112^, ^113^, ^114^, ^129^]

## 3 LDHs and CO_2_ Conversion: Experimental and Computational Toolboxes

In this section, the most relevant methodologies used to investigate heterogeneous photo‐ and electro‐ catalysts are reviewed. After a brief introduction on the basics of photocatalysis, electrocatalysis and photoelectro‐catalysis, we focus both on the experimental and the computational toolboxes used by chemists for studying LDH compounds.

On the experimental side, we review the reaction setups to perform CO_2_RR. Furthermore, we provide an overview of advanced characterization techniques to study and understand (i) catalyst's structural, morphological and physico‐chemical properties useful in the photo‐ and electro‐ catalytic conversion of CO_2_, and (ii) intermediates/products detection to figure out how the catalyst works (active sites and reaction pathways) and how its structure is modified along the reaction. Therefore in‐situ/operando microscopy, vibrational and X‐ray spectroscopy techniques applicable to this aim are briefly presented.

Regarding computational methodologies, we offer a concise guide to the computational modeling strategies ‐ i. e. the cluster and the slab approaches‐ and theoretical methods ‐ i. e. molecular mechanics (MM), density functional theory (DFT), and molecular dynamics (MD) ‐ commonly employed in the studies of LDHs in catalysis.

### 3.1 The Experimental Toolbox

In the following paragraphs a general overview of theoretical basis of photo‐, electro‐ and photoelectrocatalytic CO_2_ conversion methods is presented, together with the most relevant advances in experimental setups for conducting the catalytic transformation. Further, characterization methods for studying and monitoring carbon dioxide activation and conversion processes are revised. We postpone the discussion about suitable catalytic materials driven by the need to provide a preliminary general focus on experimental toolbox. However, it is worth anticipating that LDH materials, particularly those containing copper or in combination with it have practically demonstrated advantages while used as photocatalysts and electrocatalyst for CO_2_RR in many literature works, as will be discussed later in this review within **Section** **4**.

#### 3.1.1 Photocatalytic Strategy

**Figure 3:**
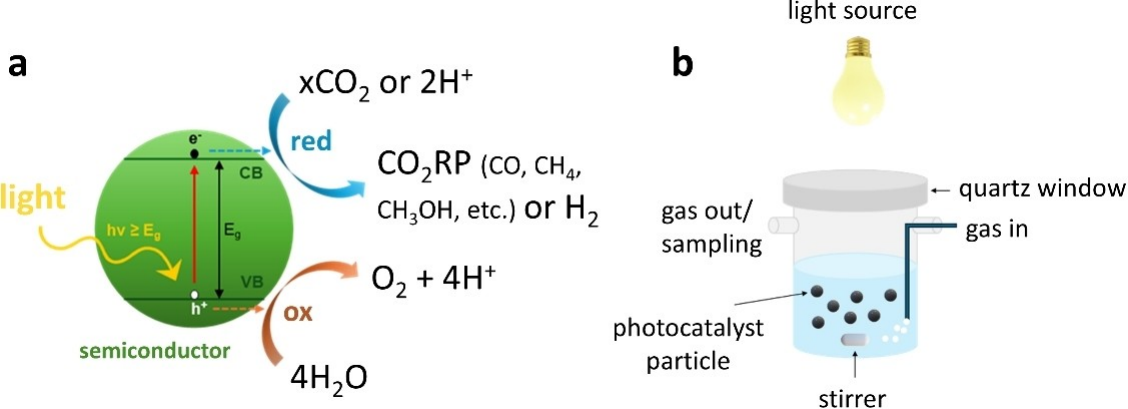
(a) Photocatalytic CO_2_ reduction reaction's mechanism over a photocatalyst surface and (b) schematic representation of slurry photo‐reactor setup (right).

Heterogeneous photocatalysts are solids, generally powders, which can accelerate a reaction upon irradiation with electromagnetic radiation, usually in the UV‐Visible range.^[139]^ These materials are usually semiconductors that create photogenerated charges (electrons and holes) upon absorption of the incident photon energy. In general, when an electromagnetic radiation (hν) with an energy that equals or is greater than the semiconductor's band gap (E_g_) interacts with a semiconductor (SC), an electron‐hole (e^−^ – h^+^) pair is created as the electron is transferred to the conduction band (CB), while a hole remains in the valence band (VB) as the electron has moved. These free e^−^ and h^+^ are highly reactive and after their migration over the semiconductor surface they can interact with external molecules driving redox reactions, as depicted in Figure 3. However, the two charges can also recombine, with the electron returning in the valence band to fill the previously generated hole. In the latter case, no reaction takes place at the surface of the semiconductor. Therefore, an optimal photocatalyst should selectively promote the reaction between the electron or the hole and an external molecule, while hindering charge recombination. Moreover, the valence and conduction bands should be located at the right energy levels to be aligned with the oxidation and reduction potentials of the interested reactions.

Photocatalytic CO_2_ conversion (PCC) to useful and value‐added chemicals (CO, CH_4_, C_2_H_5_OH, C_2_H_4_, CH_3_OH, HCOOH, etc.) is one of the most interesting approaches. This strategy is based on the concept of natural photosynthesis, employing a semiconductor material as photocatalyst. It mimics photosystem II in plants, that under light irradiation produce reducing species (e. g., from H_2_O/H_2_) which are then exploited for CO_2_ conversion to useful chemicals.^[140]^

Regarding the photocatalytic CO_2_ reduction, the minimum conduction band energy must be higher than that for CO_2_ reduction potential and the redox potential levels must be within the band gap of a chosen semiconductor material. For the reduction of CO_2_ to hydrocarbons, electrons in the semiconductor should feature a more negative redox potential, while holes must lie on the more positive potential level for water oxidation reaction. Moreover, as shown later in Table 2, a total of 2–18 electrons are required to reduce CO_2_ into hydrocarbon fuels, which is a challenging process especially compared with the hydrogen evolution from water reduction that only requires 2 electrons.[^141^, ^142^]

**Table 2:** Standard Gibbs free energy changes (ΔG^0^) and formal electrochemical redox potentials (E^0^) relative to reversible hydrogen electrode (RHE) for relevant reactions of CO_2_ reduction. Adapted from reference [158]. Copyright © 2022 Wiley‐VCH GmbH.

Reaction	Δ*G*0 [kJ mol^−1^]	Electrochemical reduction half‐reaction	*E*0 [*V*_RHE_]
H_2_O(l)→H_2_(g)+1/2O_2_(g)	237.2	2H^+^+2e‐→H_2_(g)	0.000
–	–	CO_2_(g)+e^−^→CO_2_^. −^	−1.436
CO_2_(g)→CO(g)+1/2O_2_(g)	257.4	CO_2_(g)+2H^+^ →2e^−^→CO(g)+H_2_O(l)	−0.107
CO_2_(g)+H_2_O(l)→HCOOH(l)+1/2O_2_(g)	270.4	CO_2_(g)+2H^+^+2e^−^→HCOOH(l)	−0.171
CO_2_(g)+H_2_O(l)→HCHO(l)+O_2_(g)	521.9	CO_2_(g)+4H^+^+4e^−^→HCHO(l)+H_2_O(l)	−0.071
CO_2_(g)+2H_2_O(l)→CH_3_OH(l)+3/2O_2_(g)	702.8	CO_2_(g)+6H^+^+6e^−^→CH_3_OH(l)+H_2_O(l)	+ 0.016
CO_2_(g)+2H_2_O(l)→CH_4_(g)+2O_2_(g)	919.6	CO_2_(g)+8H^+^+8e^−^→CH_4_(g)+2H_2_O(l)	+ 0.169
2CO_2_(g)+2H_2_O(l)→CH_3_COOH(l)+2O_2_(g)	963.8	2CO_2_(g)+8H^+^+8e^−^→CH_3_COOH(l)+2H_2_O(l)	+ 0.098
2CO_2_(g)+2H_2_O(l)→CH_3_CHO(l)+3/2O_2_(g)	1124	2CO_2_(g)+10H^+^+10e^−^→CH_3_CHO(l)+3H_2_O(l)	+ 0.060
2CO_2_(g)+2H_2_O(l)→C_2_H_4_(g)+3O_2_(g)	1332	2CO_2_(g)+12H^+^+12e^−^→C_2_H_4_(g)+4H_2_O(l)	+ 0.085
2CO_2_(g)+3H_2_O(l)→C_2_H_5_OH(l)+3O_2_(g)	1327	2CO_2_(g)+12H^+^+12e^−^→C_2_H_5_OH(l)+3H_2_O(l)	+ 0.084
3CO_2_(g)+4H_2_O(l)→C_3_H_7_OH(l)+9/2O_2_(g)	1962	3CO_2_(g)+18H^+^+18e^−^→C_3_H_7_OH(l)+5H_2_O(l)	+ 0.095

Beside the challenges related to the development of an effective photocatalyst with suitable physico‐chemical properties to promote CO_2_ conversion powered by photogenerated electrons, the photocatalytic reactor design plays a significant role in ensuring the highest possible performances of the process. Many parameters, indeed, can influence these performances, including the irradiation source and its position inside or outside, the reactor's material, the heat exchange and mixing, and the type of contact between the phases.^[143]^ The choice of the photoreactor for CO_2_ photoreduction (CO_2_PR) is based on three main considerations: (i) the phases involved (gas–solid, liquid–solid, gas–liquid–solid); (ii) the mode of operation i. e., batch, semi‐batch or continuous; (iii) the photocatalyst form/design, i. e. suspended or immobilized.^[144]^ The choice of the reactor can strongly affect the LDH structure, the mechanism of CO_2_‐structure interaction and the mechanism of CO_2_ conversion. Slurry photo‐reactors, in which photocatalyst’ particles are suspended in solution by stirring (Figure 3) are the most common and simplest systems in the field of photocatalytic processes. In the specific case of gas reactants, as CO_2_, the pressurization of the gas prior its injection could be exploited to provide solution mixing and photocatalyst suspension.^[145]^ However, fixed‐bed photo‐reactors, where the catalyst is immobilized on fixed supports such beads, fibres, plates, or monoliths, are usually preferred for gas‐solid reaction systems.^[143]^ Using these setups not only allows for process intensification but also helps the quantification of quantum yield and catalyst comparison. As suggested by Kisch at al. and Zani et al., light scattering phenomenon occurs when heterogenous catalysts suspended in liquid are illuminated.[^146^, ^147^] These may affect quantum yield calculation as scattered photons, if not properly counted, may be evaluated as absorbed photons. Indeed, quantum yield is calculated as the ratio between the electron molar flux involved in the redox reaction (i. e. stored or extracted by the reaction product) and the absorbed photon molar flux. Therefore, it might undergo an underestimation if the absorbed photon flux is not correctly determined taking into account the portion of scattered and transmitted photons.^[139]^ If the catalyst cannot be supported, avoiding the scattering of the suspended particles, plotting the wavelength‐dependent apparent quantum yield at different wavelengths and comparing it with the catalyst absorption spectrum is a good practise suggested by Zani et al. Nevertheless, the deposition of the catalyst on structured systems, like plates, can help to reduce scattering.

Moreover, the way in which the catalytic performances are reported and compared should be normalized.^[148]^ Usually, performances are expressed as product formation per unit mass of photocatalyst divided by the reaction time. Although this allows for comparison of data obtained with materials with the same composition and structure, it does not account for variation in the material density and surface area. Thus, the utilization of the product formation per unit of time divided by the surface area of the catalyst or even better the expression of the turnover number (TON), where the product formation per unit of time is normalized on the number of active sites are more useful values to compare catalyst coming from different synthetic methods and with different compositions and properties. As all these values include the product formation rate, they should be obtained in kinetic conditions, i. e. at low conversions, far from the thermodynamic equilibrium. A more detailed insight into photo‐reactor type and their characteristics can be found in references [143], [144], [145].

#### 3.1.2 Electro‐ and Photoelectro‐Catalytic Strategies

Electrochemical reactions involve processes of electron transfer (ET) between a solid electrode and a reactant species that must be adsorbed on its surface.^[149]^ Once the ET process is completed, the converted reactant, i. e. the product, must desorb from the electrode surface and migrate back to the bulk of the electrolyte solution. The reaction occurs at the boundary between electrode and electrolyte, also called electrical double layer. This kind of process is addressed as electrocatalysis and it is characterized by the multiphase nature of the reaction environment, where the electrode acts as solid catalyst, and the reactant is dissolved in liquid phase with the eventual copresence of a bubbled reagent gas (pure or diluted CO_2_) and/or the evolution of a product gas (e. g. H_2_). Therefore, it can be considered as a sub‐class of heterogeneous catalysis.^[150]^ The physical device in which an electrochemical reaction is performed is the electrochemical cell (EC). This hosts two complementary semi‐reactions, i. e. oxidation and reduction, also called half‐cell reactions, which occur at the surface of the two main electrodes composing the cell, i. e. anode and cathode for oxidation and reduction, respectively. Here, electrons are exchanged between the reactants within the electrolyte (i. e. ionic conductor) and the conducting or semiconducting solid electrode material. The electrons travel through an external electrical circuit, from the anode (where they are extracted from a reagent, which is oxidized) to the cathode (where they are transferred to the species to be reduced, driven by the applied voltage). Meanwhile, ions move through the electrolyte to maintain the charge neutrality. In the most simple EC the two compartments are not physically separated and share the same electrolyte solution, while in more advanced systems a barrier between the compartments, such as a “salt bridge” or a polymer membrane, allows ion conduction avoid the mixing of reactants and products of the two half‐cell reactions.^[151]^

In the case of CO_2_RR, electrons (typically extracted from water molecules oxidized to molecular oxygen at the anode) and protons are transferred to CO_2_ at the cathode. This process leads to the reduction of CO_2_ to one or more CO_2_RR products that are listed in Table 2. All CO_2_RRs in Table 2 have a positive Gibbs free energy so that an external energy input, in the form of electrochemical potential, is required for the reaction to take place.

**Figure 4:**
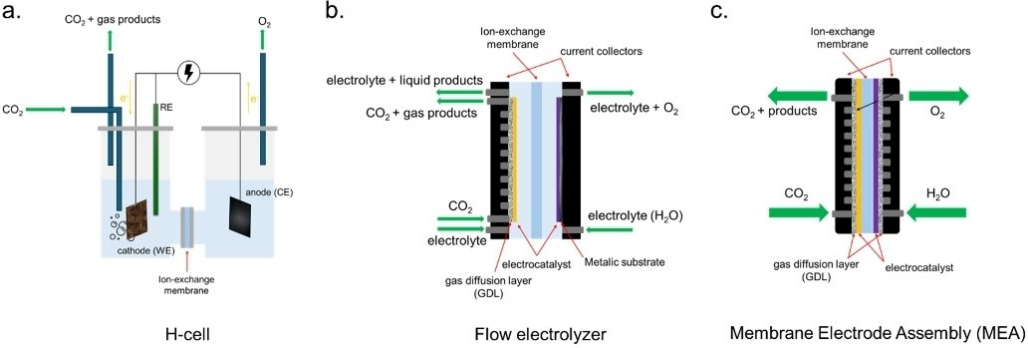
Schemes of different type of ECs for CO_2_RR: (a) H‐type cell, (b) liquid phase flow electrolyzer with gas diffusion electrode (GDE) and (c) Membrane Electrode Assembly or zero‐gap cell.

Catalytic testing can be seen as the final characterization of the electrocatalyst. Typically, the first electrocatalyst screening is conducted in batch cells with single or double compartment (H‐cells, schematized in Figure 4 a) employing noble metal (Pt, Rh, Ir) in sheet, wire, gauze form as counter electrode (anode) to speed up water oxidation thus ensuring that the reduction of CO_2_ is the rate limiting process. This allows to concentrate on the cathodic reaction where an electrode of small dimensions (around 1x1 cm or even smaller) can be used at this stage allowing rapidly testing different catalyst compositions under different reaction conditions. Nevertheless, some care has to be taken to provide a thorough characterization of the catalytic activity. For instance, the production of bubbles of products such as hydrogen may affect the availability of the catalytic surface for the reaction hindering electrolyte's ions and reactants diffusion toward electrode surface or may lead to catalyst detachment and thus deactivation. Moreover, the product‘s nature may affect the pH of the electrolyte in case carboxylic acids and/or hydroxyl ion from water are formed. As the product is generated over the catalytic site, the local pH changes and may lead to catalyst dissolution, though a detectable variation of the pH of the bulk solution may not be observed. Though analysis of traces of the components of the catalysts by Inductively coupled plasma atomic emission spectroscopy (ICP‐AES) may help to determine eventual catalyst dissolution.^[152]^ The pH is also affected by the nature of the employed electrolyte.^[153]^ The change in local pH can also favour the unwanted hydrogen reduction reaction, which is pH sensitive, and change the product distribution. In addition, the use of different electrolytes may affect catalyst stability and activity as both anion and cation nature may interact differently with the catalyst and provide different CO_2_ solubility.

Further, another parameter that is affected by the reaction configuration is the availability of CO_2_ which must be fed to the electrolyte and can interact with the electrode as a gas or, more commonly, as dissolved CO_2_ into the electrolyte. The utilization of a CO_2_‐saturated electrolyte leads to low concentrations of reagent interacting with the catalytic electrode which does not favour industrialization. The utilization of materials, such as the LDH presented in this review, able to interact with CO_2_ and carbonates and to act as a surface concentrator (see **Paragraph 4.1**) or the use of a reactor where pure or highly concentrated CO_2_ is fed as humidified gas, can be exploited as strategies to overcome low CO_2_ solubility issue.[^154^, ^155^] In this setup, the cathodic chamber is not filled with the liquid electrolyte but only with a flow of the reagent gas (diluted or concentrated carbon dioxide that can also be humidified). To make the electrolyte available for the reaction to occur, the cathode (composed of a catalyst layered deposited on a porous support that both provide electrons and mechanical strength) is in direct contact with an ionic membrane (e. g. a Nafion membrane for proton conduction). At the other side of the membrane, the anodic compartment, filled with the desired electrolyte and the anode as well as the reference electrode is found, allowing to close the circuit and perform the reaction.

The evaluation of electrocatalytic efficiency is usually based on the Faradaic Efficiency (FE%) factor which provides a quantitative information about the catalyst's selectivity and overall electrons‐to‐products conversion of the process.^[156]^ It can be expressed as a ratio between the theoretical charge needed for the production of a defined amount of product and the overall charge passed during the reaction, following the equation below:

(1) $$FE\left(\%\right)=\frac{\eta \cdot z\;\cdot F}{Q}\times 100$$

where ɳ is the number of moles obtained for a target product, *z* is the number of electrons exchanged during the reaction to get the product, *F* is the Faraday's constant (F=96485 C mol^−1^), and *Q* represents the overall charge passed during the reaction. Therefore, the collection and proper analysis of the reaction products obtained plays a crucial role while performing a study on electrochemical CO_2_RR. However, this experimental step is not straightforward due to the presence of both gas phase and liquid phase products, the volatility of some of the latter and a large variety of products that can also be produced in traces. Thus, the analytical system must comprise a gas analyzer, typically a gas chromatograph able to detect hydrogen (co‐produced by electrolysis of water and needed in the calculation of the faradaic efficiency) as well a C_1_ product and gaseous C_2_ products. However, some products may not be detected by the available gas chromatograph and for this reason a useful setup involves the bubbling of the developed gas in a cold trap (with water or a organic solvent). The obtained liquid solution can be then analysed with quantitative ^1^H‐NMR employing deuterated water (or the appropriate solvent) and an internal standard to get further insights into the product distribution.^[37]^ The same technique along with other alternatives such high‐performance liquid chromatrograpy (HPLC), head space gas chromatography (HSGC), ion chromatrography (IC) and differential electrochemical mass spectrometry (DEMS) can be applied to the analysis of the liquid phase, helping the understanding of the catalytic activity and eventually of the reaction mechanisms.^[157]^

While traditional ECs are useful for fundamental research, for efficient and technologically competitive CO_2_ reduction more complex systems have been developed considering both reduction and oxidation reactions to generate valuable products. This implies, for example, the separation of reduction and oxidation products to avoid reoxidation of reduced compound and to achieve their outlet streams in the purest form possible. Large efforts have been done in the field of electrochemical reaction setup optimization, leading to different configurations, as reported in Figure 4 b–c.^[158]^ For instance, advanced configurations comprise flow‐cells where the electrolyte is constantly fed to the electrodes and where cathode is composed by an electrocatalyst deposited over a conductive support which can be a bulk layer or a porous substrate (i. e. gas diffusion electrode – GDE) which allows for gaseous carbon dioxide being fed from the backside of conductive support. Even more advanced systems comprise a membrane electrode assembly (MEA), i. e. a “sandwich” like structure where the two electrodes are attached to the two sides of a ionic membrane that acts as the separator, resulting in a zero‐gap cells.^[159]^ This last configuration is similar to those used in PEM electrolysis and minimizes the distance between cell components thus minimizing ohmic losses. These more advanced setups allow for testing of larger electrodes (up to 25 cm^2^), work in continuous mode, lead to a higher production of reduced species and can work at different contact times between the catalyst and the reagent allowing to tune the product selectivity.^[160]^

Some pioneering studies in this field are represented by that of Kobayashi and Takahashi which demonstrated a low‐energy cell that produced methanol from CO_2_ and H_2_ with up to 97 % efficiency at −0.1 V vs the standard hydrogen electrode (SHE), using a cation exchange membrane and a Cu/Zn/Al catalyst.^[161]^ Then, Newman et al. improved proton exchange membrane fuel cells (PEMFCs) to reduce CO_2_ and H_2_O to syngas with a CO ratio of 1 : 2 at – 2 V vs. SCE and 80 mA/cm^2^.^[162]^ Dufek et al. developed a flow cell with an Ag GDE that controlled the CO ratio by adjusting CO_2_ flow and current density, achieving efficiencies of up to 92 % at 350 mA/cm^2^.^[163]^ The nature and morphology of the electrode and the design of the reactor are crucial for enhancing CO_2_ electroreduction performance; however, significant efforts are still required to reduce the reaction overpotential at both the cathode and anode to improve overall efficiency.

While renewable electricity sources can be coupled with CO_2_ electroreduction devices, direct integration of sunlight‐driven processes could offer a more efficient approach, reducing external energy requirements for CO_2_ conversion. This strategy minimizes energy losses and can eliminate the need for grid connectivity in remote areas, allowing for direct on‐site use. Photoelectrochemical (PEC) systems combine light‐assisted energy generation with the CO_2_ reduction reaction in a single device, further enhancing energy efficiency by reducing the need for external bias through photoelectrode illumination.^[164]^ In PEC technology for CO_2_ conversion, the reactor incorporates multiple components that allow the reduction reaction. Therefore, its design is crucial to the overall efficiency and effectiveness of the process.

**Figure 5:**
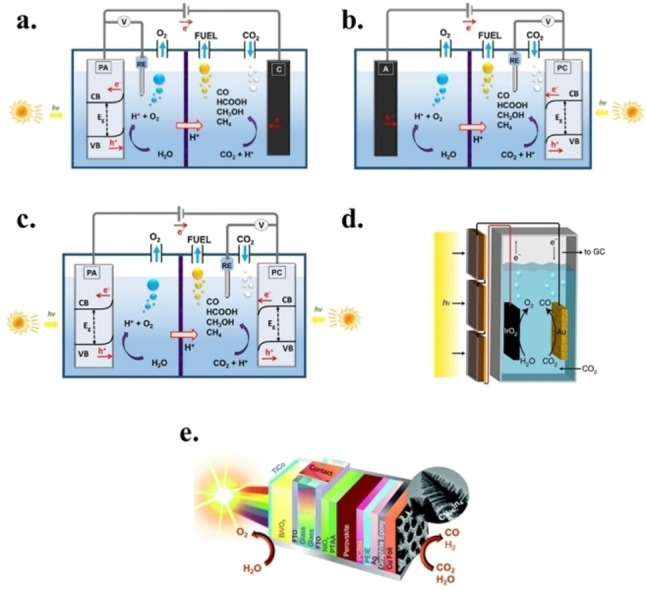
Schemes of different PEC configurations for water splitting/CO_2_ reduction: (a) photoanode‐dark cathode, (b) photocathode‐dark anode, (c) photoanode‐photocathode. Reproduced from reference [170], distributed under the terms of the Creative Commons CC‐BY license. (d) PV‐assisted electrochemical cell reproduced from reference [175], licensed under a Creative Commons Attribution 4.0 International License. (e) PV‐integrated photoanode in tandem PEC cell. Reproduced from reference [177], licensed under a Creative Commons Attribution 3.0 Unported Licence.

Similarly to classical electroreduction, PEC reactors can be divided into H‐cell type or flow type, with similar consideration respect to electrocatalytic systems in terms of advantages and disadvantages. The presence of a light source modifies the design of the H‐cells which must allocate a planar quartz window for the light irradiation of the photoelectrode(s). However, flow cells configurations up to the MEA concept have been developed.^[165]^ Depending on which electrode must be illuminated, various PEC cell configurations for water splitting and artificial photosynthesis have been explored and discussed recently.[^166^, ^167^, ^168^, ^169^] Figure 5a illustrates an example where an n‐type photoelectrode is used as a photoanode for the oxygen evolution reaction (OER), paired with a metal cathode for the CO_2_RR.[^170^, ^171^] Alternatively, the metal cathode can be replaced with a p‐type electrode, enhanced with a catalyst for CO_2_RR, to form a photocathode, which can be coupled with a dark anode, as shown in Figure 5b,^[172]^ or with a photoanode (Figure 5c) leading to a cell able to work without the need of external bias application.^[173]^ More recent and efficient systems have been developed combining photovoltaic (PV) cells an electrolyser, thus, decoupling the light‐harvesting (current generation) and the electrochemical conversion steps (PV+EC system, Figure 5d),[^174^, ^175^] or hybrid tandem electrolysers in which a solar cell is integrated with photoelectrodes, either photoanode (Figure 5e)[^176^, ^177^] or photocathode.[^178^, ^179^] All of these systems typically include (photo)electrodes, electrolytes, an external circuit, a solid separator (typically and ion‐exchange membrane), and a reactant feed. The separation of cathodic and anodic compartment allows to prevent product crossover and re‐oxidation, while also facilitating electron transfer between the cathode and anode.

The performance of PEC systems relies heavily on the efficiency of photoelectrodes and reactors in generating, separating, and injecting charges for CO_2_ reduction. However, scaling PEC CO_2_ reduction to an industrial level faces challenges, particularly the high external voltage (overpotential) required to drive the reaction efficiently. While the goal is to achieve a bias‐free PEC electrolyser, several constraints must be addressed.^[180]^ One limitation of CO_2_ reduction is the complexity of intermediates and reaction pathways, which reduces reaction selectivity and necessitates product separation. High‐selectivity (photo)catalysts, along with optimized (photo)electrode morphology, electrolyte composition, and reactor configuration, can improve system efficiency. Among the strategies to optimize photocathodes activity in harnessing light, effectively generate free‐charge carriers, and catalyse CO_2_ reduction are materials doping, defect engineering, surfaces nano‐structuring, cocatalyst integration, heterojunction formation.^[141]^ An open challenge is the development of efficient flow cells with a suitable interaction of light on membrane electrode assembly.^[181]^

#### 3.1.3 Understanding of Catalytic Phenomena

**Figure 6:**
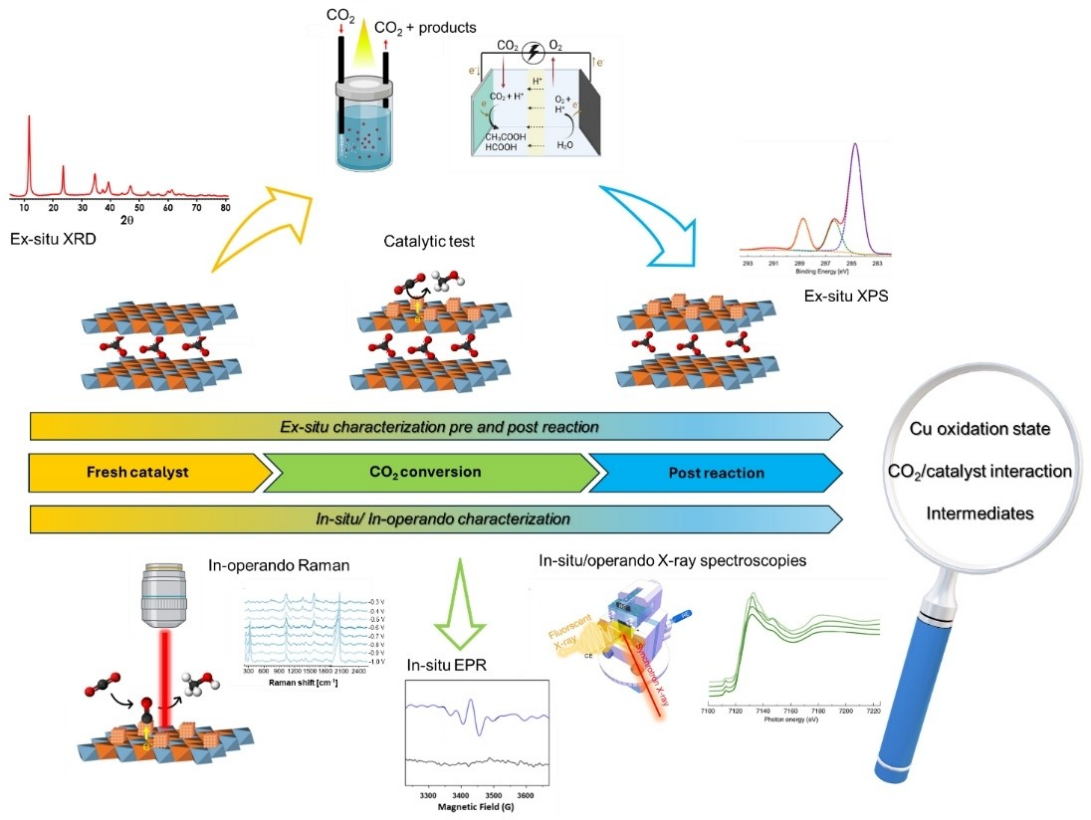
Graphical representation of experimental research steps and tools for understanding CO_2_ photo‐ or electro‐ catalytic phenomena occurring with copper‐based catalyst.

The photo‐, electro‐ and photoelectrocatalytic processes of CO_2_ reduction presented herein are characterized by several possible reaction pathways and associated rate‐limiting steps. The latter depends on the type of CO_2_ streams that need to be converted, which affects catalyst structure leading to significant changes under realistic working conditions. As a consequence, conflicting results regarding the active sites in the precursor and the real CO_2_ reduction mechanism were reported.^**[182,183]**^ This is particularly true for Cu_x_O‐based catalysts, because they may undergo dynamic structural changes during the photocatalytic CO_2_ reduction,^**[184]**^ including photocorrosion phenomena (e. g.., self‐reduction or self‐oxidation caused by electrons photogenerated *in‐situ*) upon prolonged illumination^**[185]**^ as well as partial reduction when high cathodic current flows through in electrocatalytic process.^**[186]**^ Therefore, it is essential, even if challenging, to combine reaction tests catalytic runs with, off line techniques and *in situ* and *operando* characterization techniques under experimental conditions to monitor the dynamic evolution of the catalysts and reaction products/intermediates in real‐time, as summarized in Figure 6.

Despite that, when sophisticated *operando* cells are not available, useful insights can also be obtained by means of more conventional and easily performed *ex situ* characterizations before and after catalytic runs. Even though these traditional *ex situ* characterization techniques are less attractive in the context of coupled experimental‐computational methods, some key aspects specific to the challenge of a Cu based CO_2_RR are worth mentioning. For instance, XRD characterization before and after reaction is largely employed as the most fundamental mean to assess the stability of LDH materials under operative conditions, because the decomposition of the parent LDH‐structure with segregation of CuO_x_ phases can be easily detected;[^37^, ^187^] moreover, within certain limits (e. g., if the reduction is of copper occurs extensively and involves the bulk of the material with segregation of CuO_x_ phases or metallic Cu) X‐ray diffraction allow to distinguish between Cu(II), Cu(I) and Cu(0) species.^[188]^ On the other hand, when reduction/reconstruction phenomena are concentrated within a few nm thick layer (and thus limited to the surface of the material) the XRD usually fails to give insights into these phenomena. Therefore, during the last few decades, photoelectronic spectroscopy techniques such as X‐ray photoelectron spectroscopy (XPS) and Auger spectroscopy became more and more common, ultimately becoming a standard *ex situ* characterization technique as much as XRD. Among the useful information obtainable by means of XPS is worth mentioning the determination of relative amount of the different elements at the surface of the material, as well as the ratio among the reduction state of Cu species induced by the interaction with reactants in relevant reaction conditions; in fact, the XPS binding energy (BE) of the characteristic core‐level of Cu is affected to a certain degree by its oxidation state making possible to distinguish between Cu(II) and reduced Cu in most cases, but not between Cu(I) and Cu(0) due to the overlapping binding energies;^[189]^ the determination of the ratio between the latter usually require combining XPS with Auger spectroscopy to distinguish between Cu(I) and Cu(0).^[189]^ Despite both techniques gives information about the surface of the sample (a few nm thick layer), depth profiling can be realized by combining etching/ablation techniques to XPS and Auger.^[190]^ Regarding the imaging and determination of the actual material morphology at the nanoscale, transmission electron microscopy (TEM) remains the most important *ex situ* characterization technique. In fact, it can be used to determine if the layered structure of the parent‐LDH is retained (even in the form of nanodomains) and if the reaction conditions have determined changes in phase, crystal size, or nanocrystalline domain.^[191]^ Despite this technique is extensively used as the primary method to determine the nanoparticle size distribution of supported metal catalysts (and therefore can be used to determine the particle size distribution of segregated Cu(0) or CuO_x_ after catalytic runs),^[192]^ two main challenges have to be faced as far as Cu‐based materials are concerned: the former is that small Cu(0) nanoparticles are prone to reoxidation with air even at ambient conditions, which makes more difficult to obtain representative samples with the *ex situ* approach due to the more difficult sample handling; the latter is that depending on the other elements present in the parent LDH it may be difficult to distinguish Cu nanoparticles segregated from other elements with very similar electron densities (e. g., Fe, Zn, Ga etc.).^[193]^

Solid state nuclear magnetic resonance (SS NMR) spectroscopy is a powerful tool too, providing structural information at the atomic level; it is therefore an ideal approach to obtain insights in the short‐range order of LDHs, especially considering that ^1^H, ^27^Al, ^17^O, ^13^C, and ^25^Mg NMR spectroscopy can all be used to explore the structure of LDH. However, ^17^O and ^25^Mg NMR investigations are very challenging and rarely applied, and most results involve *ex situ* studies with ^1^H, ^27^Al, and ^13^C. Nonetheless, this method was recently used to elucidate complex structural phenomena such as the mechanism of the so called “memory effect” of LDH in the solid state (i. e., without the involvement of liquid water),^[194]^ and to describe how the Jahn‐Teller effect of Cu can induce changes in the coordination number of Al, which is present in both octahedral and tetrahedral coordination in CuZnAl‐LDH.^[195]^

Lastly, whenever the determination of certain Cu species was not possible with the previously mentioned microscopic and spectroscopic methods, also *ex situ* titration methods have been proposed, as reported in the work of Serafini et al., where Cu/Cu_x_O‐CuMgAl LDH catalyst was solubilized through acidic digestion with an oxidant acid (i. e. HNO_3_) to dissolve all copper species while a non‐oxidant one, such H_2_SO_4_, led to the dissolution of just the oxidized copper species Cu(I) and Cu(II), allowing the quantification, by means of ICP‐AES analyses, of the ratio between metallic copper and oxidized copper species.^[37]^ Another example of such analytical strategy is the so called reactive frontal chromatographic method, in which the ratio between Cu(0)/Cu(I) is calculated by the selective oxidation of the surface metallic Cu(0) to Cu(I) with pulses of N_2_O at near‐ambient temperature, followed by reduction at moderate temperature of the Cu(I); the initial amount of Cu(I) is then determined by difference.^[196]^ Apart from ex situ characterization techniques, model reaction conditions can be used to isolate different steps of the reaction mechanism: for instance, the interaction of CO_2_ with LDH can be studied in flow or stationary condition by purging CO_2_ at different partial pressures and moisture content while using an IR cells to detect and identify the type of adsorbed species, while the strength and reversibility of the adsorption can be assessed by temperature programmed desorption (TPD) experiments.^[197]^ Intermediates or by products can be fed to the LDH as well to investigate their interaction with the active phase.^[198]^ Similar techniques can be used in electrochemical and photo ‐electrochemical cells to study evolutions with applied lights and/or electrical bias considering if a specific cell for *in situ* or *operando* experiments is not available, it should be properly designed.

While the CO_2_RR processes discussed in this review differ for the operational conditions, especially for the energy source powering the transformation, i. e. light, electrical bias or both, the pool of *in situ* and *operando* characterization techniques available to deeply investigate the dynamic evolution of reacting systems is the same. Spectroscopy and microscopy techniques play a crucial role in understanding the dynamics of catalytic processes under realistic conditions allowing researchers to observe changes in the catalyst‘s structure and electronic properties in real‐time, providing insights into the mechanisms of CO_2_RRs and the performance of various catalysts.^[199]^ *In situ* and *operando* characterization techniques encompass a range of methodologies widely discussed and reviewed elsewhere, including X‐ray Photoelectron Spectroscopy (XPS), X‐ray Absorption Spectroscopy (XAS), Raman and infrared vibrational spectroscopies, Transmission Electron Microscopy (TEM) and Atomic Force Microscopy (AFM). These techniques enable monitoring the catalyst surfaces and interfaces during the CO_2_RR, revealing critical information about the reaction intermediates and the evolution of the catalyst‘s active sites. For instance, Liang et al. highlighted the importance of techniques like scanning probe microscopy (TEM and AFM) and X‐ray characterization methods (XPS and XAS), in tracking surface/interface changes during photoelectrocatalytic CO_2_RR, emphasizing their potential to enhance our understanding of the behavior of catalysts.^[200]^ Similarly, Mu et al. discussed how *in situ* characterization as TEM, XRD, XPS and XAS can elucidate the dynamic changes in photocatalysts, while in situ surface species and intermediates can be probed by in situ Electron Paramagnetic Resonance (EPR), Fourier Transform Infrared (FTIR), and Raman spectroscopies, thereby aiding in the design of more efficient materials for CO_2_ conversion.^[201]^ Therefore, the mechanistic understanding of CO_2_RR has been significantly advanced through the application of *in situ* techniques. Further, Yalavarthi et al. discussed the use of time‐resolved Transient Absorption Spectroscopy (TAS) to study the optical properties and the dynamic processes occurring in the timescale of nano‐ to femto‐ seconds providing insights into the kinetics of electron transfer and the formation of reactive intermediates.^[202]^

The work of Hasa et al. summarized recent progress in utilizing these techniques to probe the heterogeneous electrochemical reduction of CO_2_, noting that they provide insights into the reaction pathways and the role of various intermediates.^[203]^ This is complemented by Li et al., who systematically reviewed the detection modes of *in situ* techniques and their application in monitoring catalyst evolution during CO_2_ reduction.^[199]^ The combination of experimental observations with theoretical calculations – which will be reviewed later – has proven particularly effective in elucidating the reaction mechanisms involved in CO_2_RR.[^204^, ^205^] Moreover, the integration of synchrotron‐based techniques has opened new avenues for operando studies of photo‐electrocatalytic systems. Soldatov et al. discussed the use of synchrotron X‐ray spectroscopies, including XAS, X‐ray Absorption Near‐Edge Structure (XANES), Extended X‐ray Absorption Fine Structure (EXAFS), to investigate the local atomic and electronic structure dynamics during CO_2_RR, emphasizing the importance of understanding the catalyst‐electrolyte interface.^[206]^ This approach allows for the real‐time observation of changes in the electronic states of catalysts, which is critical for optimizing their performance. Additionally, Carbonio et al. provided an overview of operando XPS and XAS techniques, detailing their advancements and applications in studying electrochemical interfaces under working conditions.^[207]^ The choice of materials for photo‐electrocatalytic applications also significantly influences the efficiency of CO_2_ reduction. Chen et al. explored the potential of two‐dimensional materials in this context, highlighting their unique properties and the necessity for in situ characterization to fully exploit their capabilities.^[208]^

In summary, *in situ* and *operando* characterization techniques are rapidly establishing as indispensable tools for advancing the field of photo‐electrocatalytic CO_2_ reduction. They provide critical insights into the mechanisms of catalytic processes, the evolution of catalyst structures, and the dynamics of reaction intermediates. As research continues to evolve, the combination of innovative materials and advanced characterization methods will likely lead to significant improvements in the efficiency and selectivity of CO_2_RR technologies. Table 3 summarizes some of the most interesting and useful *in situ/operando* techniques with related advantages and drawbacks. Examples of their application to LDH compounds will be discussed in the following sections.

**Table 3:** List of *in situ* and *operando* characterization techniques with relative advantages and drawbacks used in photo‐, electro‐, and photoelectro‐catalytic CO_2_RR studies.

In situ technique		Information		Drawbacks
Raman spectroscopy		Detects vibrational modes of molecules, providing information about CO_2_, intermediates, and products.		Fluorescence interference. Requires enhanced signals for weakly scattering species.
Infrared spectroscopy		Probes vibrational frequencies to identify intermediates like CO or bicarbonates.		Limited penetration depth (surface‐sensitive). Absorption by water can obscure CO_2_ signals.
Electron paramagnetic resonance (EPR)		Detects unpaired electrons, providing information about radical species, transition metal ions, and reaction intermediates.		Requires paramagnetic species. Limited to species with sufficiently long lifetimes to be detected (microseconds or longer). Complex interpretation of spectra, especially in heterogeneous systems or multi‐spin environments.
Transient Absorption Spectroscopy (TAS)		Monitors the changes in absorption of a material or reaction system after excitation by a short light pulse, capturing the dynamics of excited states and transient species.		Requires optically active species. Overlapping spectral features can make the identification of intermediates challenging. Complex instrumentation and analysis, especially for femtosecond‐resolution setups.
Transmission electron microscopy (TEM)		Atomic‐resolution imaging of catalysts that can reveal catalyst changes during reactions.		High‐energy electron beams may damage sensitive samples. Operando setups are complex and costly.
Atomic force microscopy (AFM)		Measures surface topography and forces, providing nanoscale insights.		Limited to surface properties. Time‐consuming for dynamic processes.
X‐ray absorption spectroscopy (XAS)		Provides local structural and electronic information about active sites.		Requires synchrotron radiation sources. Challenging to interpret data for complex systems.
X‐ray photoelectron spectroscopy (XPS)		Analyzes oxidation states and elemental composition at surfaces.		High vacuum requirements may hinder operando studies. Limited to near‐surface analysis.

### 3.2 The Computational Toolbox

#### 3.2.1 Surface Modelling: the Cluster and the Slab Supercell Approaches

Computational atomistic modeling of catalytically active surfaces tackles the challenge of determining the nuclear and electronic structures of a complex material, such as exposed surfaces. In fact, modeling is limited to a subset of atoms ‐ generally only a few hundred ‐ as the computational cost increases dramatically with the number of atoms. When dealing with surface phenomena, like in the case of heterogeneous catalysis, two main modeling approaches are commonly used: the cluster and the slab supercell methods.^[209]^ These approaches feature distinct advantages and drawbacks, making their application system‐ and problem‐dependent.

**Figure 7:**
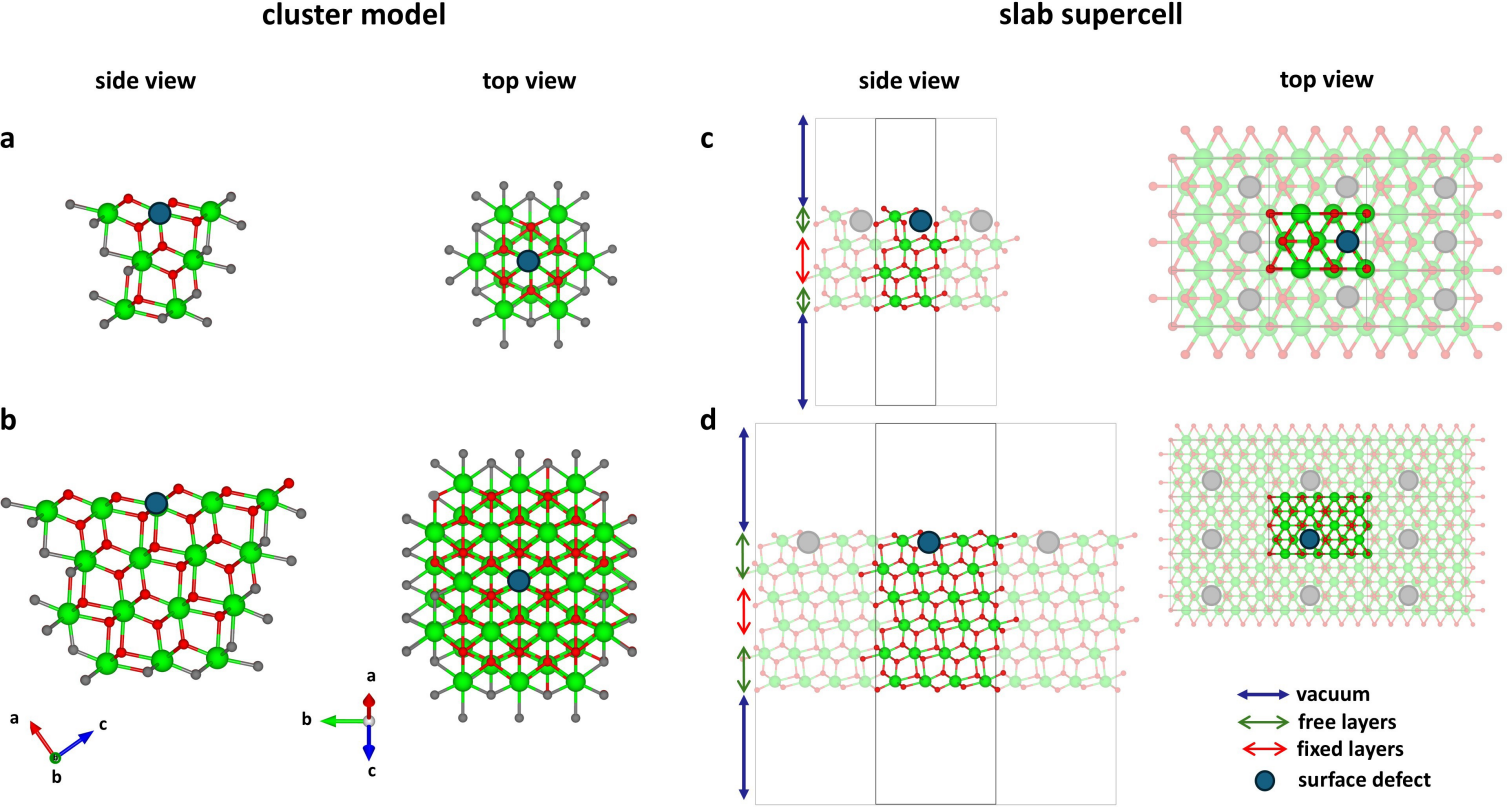
Representative cluster (a‐b) and slab supercell (c‐d) models for generic metal oxide. A smaller (a) and a larger (b) sized cluster are depicted, showing typical dangling bonds (in grey balls and sticks) resulting after extraction from the material. A generic surface defect (in blue) is added to both cluster models, showing how the clusters are generally built around the site of interest. A (1x1) smaller (c) and a (2x2) larger (d) slab supercell for the same generic metal oxide, showing the vacuum along the direction normal to the surface plane, an example of layers fixed during geometry optimizations and featuring a generic surface defect (in blue). Their periodic (first neighbour) replicas are depicted with partially desaturated colours, showing how separating local defects in the slab supercell approaches requires increasing the slab size.

In the cluster approach, a finite‐sized fragment (i. e. the cluster) is cut from the material (see Figure 7a, with examples for a simple metal oxide). The cluster can be treated as either an isolated system or embedded in a surrounding medium that mimics (implicitly) the properties of the rest of the infinite system. Therefore, clusters are advantageous for studying spatially localized phenomena, such as localized defects (see Figure 7a–b) or quantum confinement effects. This approach fits well with cases where surface periodicity is less relevant and local properties dominate in one (or more) step(s) of the surface reaction.^[209]^ In fact, the cluster approach can treat more ‘naturally’ low‐coordinated and defective sites localized on an extended surface. Clusters are generally relatively small in size and their properties can be routinely computed with quantum mechanics (QM) software generally used for molecular systems, also employing localized (typically gaussian) basis set functions.^[210]^ Moreover, thanks to the restricted number of atoms, also more advanced *ab initio* approaches, such as wave function methods, can be used to compute the electronic structure of cluster models at a very high level of theory, while describing the surrounding medium at a lower level of theory, if required. Remarkable examples are computational studies involving excited states or localized electronic effects, which greatly benefit from wave function methods.[^211^, ^212^, ^213^] As simple in theory as challenging in practice, the cluster approach is limited by two main factors. First, the size of the cluster influences the quality of the results: the treatment of very large clusters requires lowering the level of theory but too small clusters would suffer in reproducing the key electronic properties of the extended material, like the band gap.[^214^, ^215^] This issue is particularly critical for semiconductors, like LDHs, where clusters often overestimate band gaps.^[216]^ In the case of metals, the cluster “localization” can lead to an enhanced and unreliable chemical reactivity.^[217]^ Another limiting factor is that, once the cluster is cut from the material, the so‐called *dangling bonds* associated with low‐coordinated atoms at the periphery of the cluster are introduced (see Figure 7a–b). *Dangling bonds* impact negatively on the stability of the material and lead to structural deformations when the atomic structure of the cluster is optimized fully, i. e. without constraints. This issue can be mitigated by either embedding the cluster in a surrounding[^218^, ^219^] or saturating *dangling bonds* with capping atoms (generally H)[^220^, ^221^, ^222^] to ensure stability and a reliable structural description.

**Figure 8:**
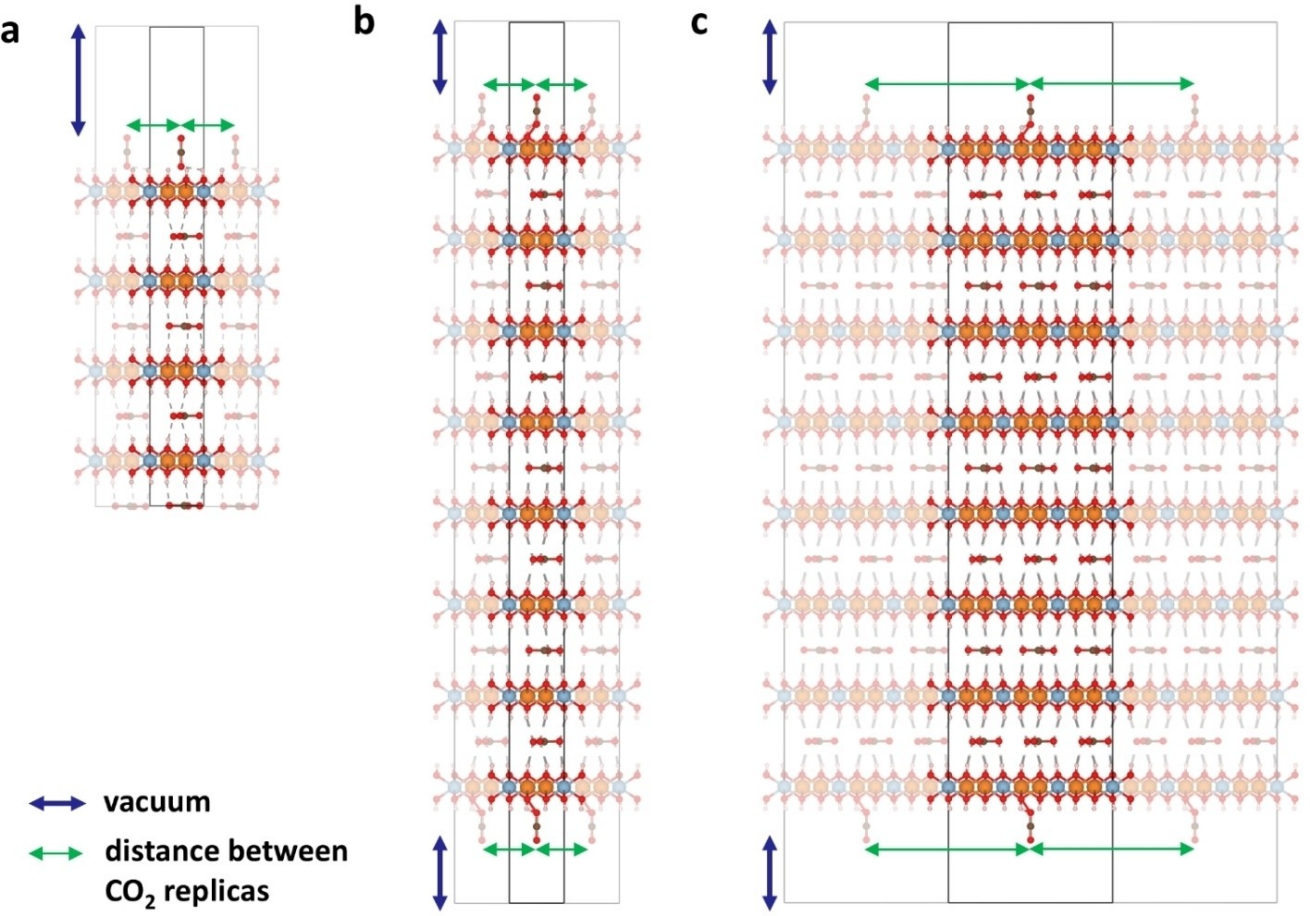
Slab supercell models illustrating CO_2_ adsorption on an LDH material. M^2+^ ions are shown in blue, M’^3+^ ions in orange, and carbonate is present in the interlayer. The slab configurations can be either (a) asymmetric or (b, c) symmetric. Transitioning from (b) a (1×1) to (c) a (3×3) supercell the distance between the adsorbate and its periodic replicas along the surface increases, minimizing interactions.

The main limitations of cluster models are surmounted by the slab supercell approach, which employs periodic boundary conditions (PBC) to replicate a unit cell, named supercell, infinitely along all crystallographic directions. Here, while the supercell is still composed by a finite (and reasonably small) number of atoms, the chemical bonds at its periphery are preserved thanks to the PBC (see Figure 7c–d). This method is the most used for modeling materials not only for bulk properties but also for surfaces, since the supercell can contain a slab of the material interfacing a (sufficiently large) vacuum regions in the direction perpendicular to the surface that one wants to model,^[217]^ as shown in Figure 7c–d. In this way the surface is exposed, and the interaction between the replica along the direction perpendicular to the surface plane is minimized. The slab approach inherently accounts for electronic delocalization (along the exposed surface plane, see top views of Figure 7c–d), providing a more accurate description of the electronic structure of an extended surface compared to the cluster model. However, the slab supercell approach also suffers from limitations. The accuracy of the slab model is sensitive to the number of layers included in the model, with the addition of more layers determining a relative increase of the computational costs. The outermost layers of the slab mimic the exposed surfaces, while the innermost layers, typically fixed during optimization, serve to represent the bulk.^[216]^ Since the slab is exposed on two sides, some issues can arise when asymmetry of the slab is present, and the exposed surfaces should be treated with caution in these cases (see Figure 8). Often, when symmetry issues are not relevant, the bottom layers are fixed during the geometry optimization and only the top layers are relaxed and used to simulate the properties of the surface of interest. Moreover, the PBC along the exposed surface plane should be set to avoid the interactions between molecules belonging to adjacent slabs if such interactions are not desired. This is, for instance, the case of low surface coverages of adsorbed molecules, when a sufficiently large surface area must be modelled, imposing a large slab and, consequently, high computational cost.

Figure 8 exemplifies the relevant case of LDH surfaces and adsorption of CO_2_. This example, indeed, reveals some limitations of the slab supercell with respect to the cluster model, since the former might require large computational efforts to model local structures and properties on surfaces. The implementation of the slab supercell approach relies on software specifically designed for periodic systems and the description of long‐range periodicity in crystalline materials. Notable examples include software based on plane‐wave basis set, such as VASP^[220]^ or Quantum Espresso,^[223]^ or gaussian localized basis set, such as Crystal,^[224]^ or on both, as in CP2 K.^[225]^ The use of wavefunction methods is not standard for these codes, which generally adopt the DFT methods that will be described below. Despite some limitations, periodic models can achieve remarkable accuracy for structural and electronic properties, making them highly effective for simulating surface interactions and catalytic processes.^[216]^ Our group, in fact, has successfully employed both cluster and slab supercell approaches to study the adsorption of small molecules and organic compounds and chemical reactions on metallic, bimetallic and oxides surfaces.[^226^, ^227^, ^228^, ^229^, ^230^, ^231^]

The cluster and slab supercell approaches define the structural models of the material, but not the levels of theory that can be employed, which are reviewed in the following sections. In particular, classical MM^[232]^ and quantum‐based DFT^[233]^ theories are briefly outlined, as these are the most common methodologies used for studying LDHs materials. Since a full description of QM methods is much beyond the scope of this review, we assume that the reader is familiar with the basic ideas of QM and the Hartree‐Fock theory.[^234^, ^235^]

#### 3.2.2 Molecular Mechanics

MM is a method based on a balls‐and‐springs description of atom interactions. Namely, MM employes classical potential functions to describe the forces acting in a molecular system. The overall potential energy accounts for several terms as follows:

(2) $$\;E_{total\;}=E_{bond}+E_{angle}+E_{torsion}+E_{vdW}+\;E_{electrostatic},$$

where E_bond_ accounts for the energy associated with the stretching or compression of bonds; E_angle_ describes the energy change when bond angles deviate from their equilibrium values; E_torsion_ accounts for the energy associated with rotation around a bond (i. e. torsion angle); E_vdW_ models the attractive and repulsive forces between non‐bonded atoms; E_electrostatic_ describes the Coulombic interactions between charged particles. The whole model depends on both geometrical parameters and constant terms. Different MM models mainly differ for the inclusion of extra‐terms, e. g. anharmonic corrections, and the so‐called force field (FF), which defines the current values of constants, i. e. non‐geometrical terms, determining each term of **Equation** **2**. The accuracy of MM‐based calculations mainly relies on the choice of the FF, which depends on the atoms and the chemical properties involved, thus is system‐dependent. Examples of FFs applied to material science are the Embedded Atom Model (EAM) for metal and alloys,^[236]^ the Tersoff^[237]^ and the Stillinger‐Weber^[238]^ for covalent materials, and the ClayFF for clays like materials,^[239]^ such as LDHs.

MM simulations benefit from exceptional computational efficiency, which allows for exploring extended materials and their dynamical effects, when paired with MD approaches simulations, as described later. However, traditional MM implementations do not provide models for accurately describing reaction mechanisms, which usually require QM methods that describe the changes in electronic structure due to chemical reactions. More recently, reactive FFs have been developed to account also for chemical transformations, like ReaxFF,^[240]^ but to the best of our knowledge have not been applied yet to LDHs.

#### 3.2.3 Density Functional Theory

Despite the advancements of wave‐function based methods, applications to extended materials and heterogeneous catalytic interfaces remain challenging and restricted to small cluster or embedded models. In material science and computational catalysis, DFT is the most used method thanks to its gratifying accuracy at a limited computational cost, which allows to simulate systems comprising even hundreds of atoms and still retrieve correlation effects.^[241]^

The core concept of DFT is to reformulate the Schrödinger equation by replacing the full‐electron wave function with the ground state electron density as the primary variable. Namely, in DFT any ground state property is defined in terms of the electron density of the system rather than tis wave function. This paradigm shift turns into a significant computational speed‐up while still accounting for electron correlation that is crucial for accuracy in QM computations.^[233]^

DFT relies on two theorems published by Hohenberg and Kohn (HK) in 1964,^[242]^ which states that the ground‐state density uniquely determines the system‘s properties, and the energy (along with any other property) is a functional of the electron density. While the HK theorems provide the fundamental theoretical basis for DFT, the key step through a real implementation of DFT was done by Khon and Sham.^[243]^ Khon and Sham provided a DFT formalism based on the concept of a system of non‐interacting electrons that reproduces the same ground‐state electron density as the interacting many‐electron system. This allowed separation of energy contributions, reformulating the total energy functional, E[ρ] as:

(3) $$\;E\left[\rho \right]=T_{S}\left[\rho \right]+V_{ext}\left[\rho \right]+J\left[\rho \right]+E_{XC}\left[\rho \right]$$

where ρ is the electron density of the system; T_S_[ρ] is the kinetic energy of the non‐interacting system; V_ext_[ρ] is the potential energy from external fields (e. g., nuclei‐electron interaction); J[ρ] is the classical Coulomb (Hartree) energy of electron‐electron repulsion; and E_XC_[ρ] is the exchange‐correlation (XC) functional, capturing quantum mechanical exchange and correlation effects. The functional form of each term in **Equation** **3** is known, except for E_XC_[ρ], thus the efforts in the field still focus on improving the description of the XC functional,^[244]^ which highly influences the accuracy of DFT calculations.^[245]^ The simplest methods for defining E_XC_[ρ] are based on the Local Density Approximation (LDA), which assumes a uniform distribution of the electron density, which works well for systems where the electron density is almost uniform, such as metals and some simple solids. On the contrary, LDA functionals perform poorly for exposed surfaces and systems with highly inhomogeneous electron densities, such as complex solids or molecular systems. LDA often predict too strong (and too short) bonds and short lattice constant.^[246]^ The Generalized Gradient Approximation (GGA) improved upon LDA by incorporating the gradient of the electron density into the XC functional, allowing for a better description of systems with inhomogeneous densities. Among GGA functionals, the Perdew‐Burke‐Ernzerhof (PBE)^[247]^ one and its re‐parameterizations (e. g. the revised PBE^[248]^ and the PBEsol^[249]^) are very popular choices for materials, especially for surface modeling,^[217]^ thanks to its balance between accuracy and computational cost.^[246]^ Despite its tendency to underestimate band gaps, PBE performs better than LDA functionals in predicting cohesive energies and lattice constants.^[250]^ Moreover, although PBE often overestimates the dipole moments of isolated molecules, it provides reliable predictions of work functions of surfaces (discussed in more details below) for systems with polar adsorbates,^[216]^ which are pivotal in catalysis.

Further advancements in XC functionals include hybrid functionals, which combine semi‐local functionals (such as LDA or GGA) with a fraction of the exact exchange derived from Hartree‐Fock theory. Hybrid functionals overcome limitations of semi‐local functionals in the underestimation of band gaps, improving accuracy by reducing self‐interaction errors and retrieving better electronic correlation. Our group has, indeed, extensively used hybrid XC functionals to study the mechanism of activation of small molecules (including CO_2_, CH_4_, H_2_O, O_2_) in gas‐phase or in solution.[^251^, ^252^, ^253^, ^254^, ^255^, ^256^, ^257^] Further improvements of XC functionals involved the introduction of the so‐called double‐hybrid functionals, which add to hybrid functional a second‐order perturbation correction for non‐local correlation effects. However, the computational cost of hybrid and double‐hybrid functionals is significantly larger compared to GGA ones, making them less appealing for large systems, although the number of papers using hybrid functionals for extended materials is naturally growing with the improvement of high‐performance computing resources.^[258]^ For strongly correlated systems with localized electrons, such as transition metal oxides or rare‐earth compounds, the DFT+U method provides a cost‐effective approach. Based on the Hubbard model, this approach introduces additional correction effects for on‐site electron interactions in specific orbitals using two parameters: U (Coulomb interaction) and J (exchange interaction).^[259]^ These parameters can be determined semi‐empirically by either fitting experimental data or through *ab initio* methods.^[260]^ Fine‐tuning of U and J is essential for having accurate results, especially considering that they strongly depend on both the property under investigation and the local chemical environment. This makes, thus, challenging the use of DFT+U in systems with oxidation state changes or mixed valencies.

LDA, GGA, and hybrid functionals focus on the local electron density and lack to retrieve long‐distance correlation effects, like the van der Waals (vdW) interactions. These are crucial in organic systems, layered materials with intercalated molecules, or hybrid organic‐inorganic systems like catalytic interfaces. To recover (part of) the long‐distance correlation, vdW functionals can be used.^[261]^ They explicitly include non‐local correlation contributions within the XC functional. While this approach can be more accurate, it is computationally expensive. A more computationally efficient option is the use of *a posteriori* correction method. These methods compute vdW energies separately, after the self‐consistent cycle has converged, using an analytical expression based on pairwise interactions. Examples include the widely used Grimme DFT−Dx methods^[245]^ and Tkatchenko‐Scheffler approach.^[262]^ These corrections are computationally cheap and have been successfully applied to various systems, offering a practical way to account for vdW interactions without significantly increasing the computational burden.^[216]^

To summarize, we provided a short overview of the fundamentals of DFT indicating the state‐of‐the‐art implementations that are generally adopted for studying materials like LDHs ‐ i. e. the use of PBE functionals, the inclusion of non‐local correction terms, and the Hubbard correction‐, more generally representing the toolbox of the computational chemists to investigate the properties of heterogeneous materials, providing information about their structural and electronic properties.

DFT can be indeed used to compute fundamental structural parameters of a material. By minimizing the total energy of the system computed through DFT methods, the equilibrium lattice constants and atom positions in the unit cells (or slab supercells for surfaces) can be evaluated. Furthermore, DFT can be used to characterize the electronic structure of materials. By choosing a suitable E_XC_, a reliable description of the electronic band structure, which discloses the energy levels of electrons as a function of their momentum in the Brillouin zone.^[263]^ This information is crucial for distinguishing between metals, semiconductors, and insulators behavior based on the presence or absence of a band gap, E_g_, between the valence band maximum (VBM) and the conduction band minimum (CBM). The analysis of the band structure can provide the density of states (DOS), which describes the number of electronic states available at each energy level within the material, giving insights into the distribution of electrons across different energy levels.^[263]^ DOS analysis helps identify states near the Fermi level, i. e. the highest occupied energy level at 0 K, which are critical for a material's electrical conductivity and chemical reactivity. For complex materials with multiple atomic species, the projected DOS (PDOS) can reveal the contributions of individual atoms or orbitals to the electronic structure, offering insights into how specific elements influence material properties.^[264]^ This is crucial in the case of reactive surfaces, where PDOS can shed light into the interaction of the molecule with a certain active site. Finally, also the work function is an informative quantity,^[264]^ defined as the minimum energy required to remove an electron from the material‘s surface to vacuum, which gives insights into electron transfer processes and activation of molecular surface species.

#### 3.2.4 Molecular Dynamics

In material science, dynamical effects play a crucial role.^[232]^ Namely, at high temperatures or, more specifically for LDH‐like systems, in the interlayer regions of layered materials, the motions of atoms and molecules cannot be ignored as they intrinsically affect the properties of the material. MD simulations allow catching the dynamics of systems under various conditions with atomic resolution.^[265]^ In the limit of the Born‐Oppenheimer approximation, MD simulations describe the motion of atoms, i. e. the time‐evolution of nuclei's positions, according to the Newton's equations of motion as follows:

(4) $$\;F_{i}=m_{i}\cdot \;a_{i},$$

With m_i_, the mass of the *i*‐th particle and a_i_ its acceleration, given by:

(5) $$\;a_{i}=\frac{d^{2}r_{i}}{dt^{2}},$$

and F_i_ the force acting on the *i*‐th particle, also expressed as follows:

(6) $$\;F_{i}=-\nabla U\;\left(\left\{r_{i}\right\}\right),$$

with U({r_i_}) the potential energy of the system as a function of the position of particles.

Even if the motion of nuclei is described classically, the potential energy surface, U({r_i_}), can be computed at any desired level of theory. Namely, both classical and QM methods can be used to define U({r_i_}). In classical MD, the potential energy is determined using a classical FF. As for MM, this approach is suitable for studying large systems over long timescales, and with limited accuracy. In *ab initio* MD (AIMD), the potential energy is computed at QM level, typically using DFT.^[266]^ AIMD is computationally expensive, limiting its applications to small systems and shorter timescales than those achieved with classical MD. Nevertheless, it is worth noticing that recently ML‐based models have sensibly improved AIMD simulations. The combination of ML and AIMD allows for speeding‐up simulations by training on‐the‐fly accurate and system‐dependent FFs using a limited number of trajectories collected with AIMD.[^267^, ^268^] These methods pave the way for FFs with an accuracy close to QM while drastically reducing the computational cost of simulations.

## 4 Experimental Insights into CO_2_ Activation on LDHs

### 4.1 CO_2_ Adsorption on LDHs

Hydrotalcite‐like compounds have garnered increasing attention for their potential applications in CO_2_ adsorption.^[269]^ An ideal adsorbent should be cost‐effective, exhibit rapid adsorption kinetics, possess high capacity and selectivity for CO_2_, and demonstrate thermal and chemical stability over multiple cycles. The effectiveness of CO_2_ adsorption relies on the material's specific surface area and the number of accessible basic sites.^[270]^ Among the diverse materials being explored for CO_2_ capture and storage, LDHs and especially their derived mixed metal oxides stand out because of their high surface area, pore structure, and charge density.[^271^, ^272^, ^273^] CO_2_ adsorption on hydrotalcite‐like materials occurs naturally as atmospheric CO_2_ interacts with compensating ions in the interlayer space. Ishihara and co‐workers demonstrated this by intercalating ^13^C‐labeled carbonate ions into the hydrotalcite structure, which exchanged with CO_2_‐derived carbonate ions from the atmosphere within a day, illustrating natural carbon cycling.[^24^, ^274^] In addition, Di Bitetto et al. demonstrated that in LDH the exchange process with atmospheric CO_2_ proceeds through a mechanism that involves both carbonate and hydrogenocarbonate with HCO_3_^−^/CO_3_^2−^ equilibrium that is reached after a few minutes of air exposure. Therefore, the two species coexist in the interlayer of LDHs and the kinetics of exchange process was found to depend on the amount of hydrogenocarbonate.^[275]^ Preliminary studies on CO_2_ adsorption by hydrotalcite and related materials, conducted by Mao and co‐workers in 1993,^[271]^ involved varying the M^2+^/M’^3+^ molar ratio, which altered the layer charge of synthesized hydrotalcites. This compositional tuning produced a diverse range of materials with varying CO_2_ adsorption capacities (from 0.4 to 1.5 mmol g^−1^). In the same year, Tsuji and co‐workers examined various LDHs and found that the nature of the M(II) cation influenced selectivity toward CO_2_ adsorption, with a ranking of CuAl ~ ZnAl < CoAl < MgAl < NiAl, related to their thermal properties.^[272]^ Subsequent works explored the role of trivalent cations in determining structure evolution under thermal treatment for high‐temperature CO_2_ adsorption^[276]^ as well on hydrophilic and basic character for low temperature application.^[277]^ Further studies indicated that mixed oxides typically had superior sorption capabilities compared to fresh samples, influenced by experimental conditions.[^278^, ^279^] The basic research on CO_2_ adsorption through LDH and derived oxides have then opened the road for more complex industrial applications, such sorption‐enhanced reaction processes (SERPs), particularly in methane‐to‐hydrogen conversion.[^280^, ^281^] For instance, Coenen and co‐workers published findings on K‐promoted hydrotalcites for sorption‐enhanced water‐gas shift reactions (SEWGS).[^282^, ^283^] In light of these advancements, a new frontier of sorption‐enhanced reaction strategy may be represented by processes like electro‐, photo‐, and photoelectro‐ catalytic CO_2_ conversion which take advantages of LDHs and derived oxides CO_2_ adsorption properties.

### 4.2 Photocatalytic CO_2_ Reduction

Pristine LDH have been studied quite extensively to produce interesting products from CO_2_. A large number of works discussing the use of Cu‐free LDHs are present in literature, where Mg, Zn, Ni, Co are present as divalent metal cation and Al, Fe, and Cr as trivalent ones.[^39^, ^204^, ^284^, ^285^] Nevertheless, this review aims to focus on the state‐of‐art of those photocatalysts conceived to couple the peculiar catalytic activity of Cu in CO_2_RR with the enhanced interaction of LDHs structure with CO_2_. Therefore, in the following, only Cu‐based LDHs or Cu/LDHs hybrid materials will be discussed.

Ahmed et al. that applied ZnAl‐, CuZnAl‐ and CuZnGa‐LDHs to convert gaseous CO_2_ (2.3 kPa) by photoreduction.^[285]^ ZnAl pristine LDH was able to produce CO at a rate of 620 nmol h^−1^ g^−1^, but very interestingly methanol (CH_3_OH) was produced when Cu and Ga were introduced in the structure (170 nmol h^−1^ g^−1^). This was suggested to be due to specific interaction of Cu sites that enabled coupling between protons and photogenerated electrons leading to methanol formation. Morikawa et al. investigated the reaction mechanism that yields CH_3_OH from CO_2_ by EXAFS and FTIR over CuZnGa‐LDH.^[25]^ Electron mobility over the cationic layer was found to be an order of magnitude higher than in perpendicular way. Carbonate decomposition was increased when hydrogen was added in the reaction mixture suggesting its participation in the photoreaction of hydrogen carbonate which was confirmed to be an intermediate in the formation of CO and CO_2_. Interestingly, they also studied the effect of copper position by placing it in the cationic layer or in the interlayer in the form of [Cu(OH)_4_]^2−^.^[286]^ The latter provided higher conversion, which was related to the different interlayer spacing between the copper intercalated and carbonate intercalated LDH.

Other LDH materials based on Cu, Zn, Ga were studied by Wein and co‐workers, demonstrating its potential as efficient photocatalysts for the selective conversion of CO_2_ to CH_3_OH, with the interlayer space and high‐pressure conditions playing crucial roles in enhancing the catalytic performance.^[287]^ These results show that the LDHs can selectively produce CH_3_OH as the sole C‐containing product, with no detection of other products. This exclusive formation of methanol is attributed to the longer residence time of CO_2_‐derived intermediates within the interlayer space of the LDHs during the reduction steps. The methanol formation rate was significantly enhanced when the LDHs were exposed to air prior to the photoreduction tests, compared to as‐synthesized LDHs. This enhancement is explained by the liberation of the interlayer space due to the removal of 31 % of the interlayer water. Further experiments under high‐pressure conditions of CO_2_ (0.12 MPa) and H_2_ (0.28 MPa) showed even higher methanol formation rates, with improvements by factors of 255–645 compared to previous low‐pressure experiments. This suggests that the high‐pressure conditions favor the photocatalytic conversion of CO_2_ to methanol. XANES analysis under the high‐pressure CO_2_ and H_2_ conditions, also provided insights into the coordination environment of the metal ions in the LDH structure.

Guo and co‐workers presented the synthesis and application of a core‐shell structured ZnO@CuZnAl‐LDH photocatalyst, which provided high catalyst surface area, thus more CO_2_ adsorption sites, suggesting that the photocatalytic efficiency of LDHs can be further enhanced by forming proper junctions with other semiconductor materials, such as ZnO. This could help inhibiting the recombination of photogenerated electron‐hole pairs, leading to improved CO_2_ reduction performance.^[288]^

M_2_M’‐NO_3_‐LDH The incorporation of Cu_2_O over LDHs, achieved by *in situ* chemical reduction of copper present in CuZnCr‐LDH structure, proved to be an effective way to increase the photocatalytic activity.^[289]^ In fact, Cu_2_O decorated ZnCr‐LDH provided higher CO productivity than CuZnCr‐ or ZnCr‐LDHs. Cu_2_O acted as electron traps, promoting charge separation, and providing active sites for CO_2_ conversion. In another work, the synthesis of NiFe‐LDH wrapped Cu_2_O nanocubes led to the formation of a Z‐scheme mechanism in the photocatalyst which increased methane formation (0.83 μmol g^−1^ h^−1^) by 5.6 and 6.9 times compared to pristine NiFe‐LDH and Cu_2_O.^[290]^

**Figure 9:**
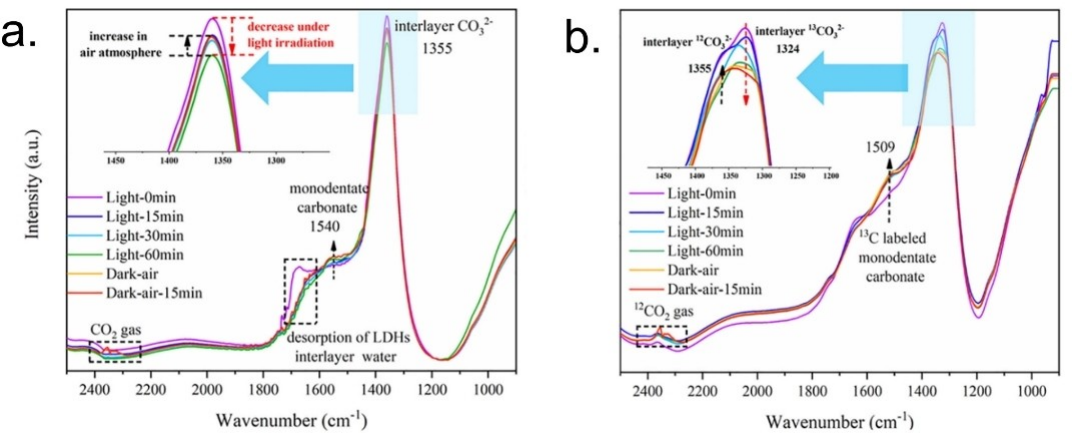
In situ FTIR for (a) ^12^CO_3_^2−^‐CuCoAl‐LDHs−E‐60 and (b) ^13^CO_3_^2−^‐CuCoAl‐LDHs−E‐60 transformation under Ar+light and air+dark conditions. Adapted from reference [291]. Copyright (2022), with permission from Elsevier.

More recently, the study of Fan et al. explored the use of CuCoAl‐LDH as an integrated system for CO_2_ direct capture and photocatalytic coupling reactions.^[291]^ The photocatalytic coupling reaction involved the reduction of CO_2_ and oxidation of 5‐hydroxymethylfurfural (5‐HMF) to produce valuable chemicals like 2,5‐furandialdehyde (DFF), 5‐formyl‐2‐furanacarboxylic acid (FFCA), and 2,5‐furandicarboxylic acid (FDCA). *In situ* characterization techniques like FTIR, Raman, and X‐ray absorption spectroscopy revealed the role of metal vacancies, hydroxyl groups, and photoinduced activation of CO_3_^2−^ in enhancing the 5‐HMF activation and CO_2_ reduction performance. This study is of particular interest because it proves that in the inert atmosphere, carbonates located in the interlayers, which could be easily supplied by CO_2_ in air, realized in situ reduction catalyzed by the metal cations on the layers, and this was coupled with 5‐HMF oxidation catalyzed by the hydroxyl sites. Furthermore, a series of *in situ* FTIR spectroscopy measurements, with assistance of ^13^C labeling characterizations, as reported in Figure 9a–b, clarified the *in situ* transformation process.

### 4.3 Electrocatalytic CO_2_ Reduction

While LDHs are recognized as highly efficient photocatalysts, few studies focus on their direct role as electrocatalysts for CO_2_ reduction. More commonly, LDHs serve as supports for electrocatalysts or as precursors for producing metal nanoparticles (NPs) or metal oxide electrocatalysts through chemical or electrochemical modifications.^[22]^ The versatility of LDHs in terms of composition, properties, morphology, synthesis, carbon dioxide adsorption and electronic conduction make them suitable materials for the production and testing of Cu‐based catalysts for carbon dioxide electroreduction.

**Figure 10:**
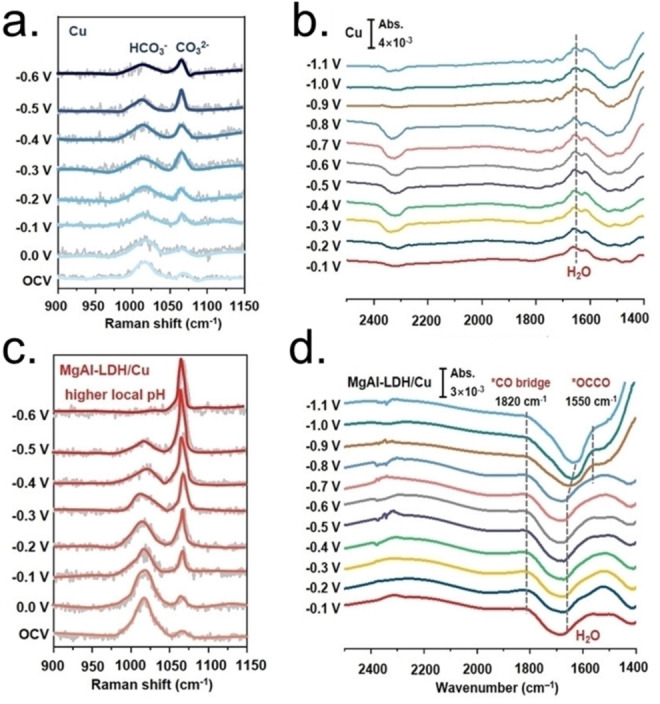
In situ characterization of MgAl‐LDH/Cu and bare Cu electrodes proposed by Xu et al. (a) In situ Raman spectra and (b) in situ FTIR spectra obtained from Cu electrodes. (c) In situ Raman spectra and (d) in situ FTIR spectra of MgAl‐LDH/Cu electrodes. Adapted from reference [292]. Copyright (2023), with permission from Wiley‐VCH.

For instance, MgAl‐LDH was shown to improve CO_2_RR performance on Cu electrodes by modifying the local pH environment, enhancing OH^−^ availability and formation of compounds with two or more carbons, i. e. C_2+_ products, particularly ethylene.^[292]^ A MgAl‐LDH/Cu electrode showed higher selectivity for C_2_H_4_ at a current density of 300 mA cm^‐2^ compared to bare Cu. Other combinations of LDH/Cu electrodes, including CoFe‐LDH/Cu and NiFe‐LDH/Cu, showed low conversion rates compared to MgAl‐LDH/Cu. XPS, EXAFS, in situ Raman and FTIR analyses agree in a local pH increase for MgAl‐LDH modified electrode respect to bare Cu electrode, due to a faster water dissociation (high concentration of OH^−^ surrounding the reaction interface), as indicated by results shown in Figure 10, thus a higher local pH is established leading to faster C_2+_ products formation.

Nevertheless, Cu can also be directly inserted as a component of LDH structure. CuAl‐LDH was first reported by Iwase et al.^[47]^ as a CO_2_RR electrocatalyst, with various samples prepared at different synthesis conditions where copper is present as the bivalent cation of the cationic layer of LDH. This electrocatalyst, tested in a gas diffusion electrode set‐up under galvanostatic conditions (50 mA), led to the production of CO, HCOOH and H_2_ as major products and ethylene and ethanol as minor ones. Li *et al*. reported a comparison between a Cu/MgAl‐ and an Au/MgAl‐LDH obtained by a co‐precipitation method.^[40]^ The respective active components (Cu and Au) were added by two different routes: Cu was added to the LDH through a pre‐intercalation of EDTA, while Au was added by an ion exchange process. Once the inks of the catalysts were obtained, they were loaded on a bulk glassy carbon electrode (1.8 cm^2^). The catalytic performances for the CO_2_RR were investigated in two different electrolytes (KHCO_3_ and alcohol amine solution, containing ethanolamine and diethanol amine). Applying a fixed potential from −0.3 to −0.5 V *vs* RHE to the Cu/MgAl‐LDH and from −0.45 to −0.7 V *vs* RHE to the Au/MgAl LDH in KHCO_3_, the authors obtained a large variety of products, including H_2_, CO, CH_4_, and HCOOH. On the contrary, upon application of a potential in the alcohol amine solution, only gaseous products were obtained, *i*. *e*., H_2_ and CO, for both LDHs.^[293]^

Other reducible cations can be also inserted into the LDH structure. Miao and co‐workers introduced Ni^2+^ into CuAl‐LDH to form a ternary CuNiAl‐LDH, resulting in a higher methanol yield, attributed to the presence of Cu^+^ and hydroxyl groups in the catalyst.^[293]^ Calcining CuNiAl‐LDH, however, reduced methanol selectivity due to structural collapse and loss of hydroxide groups. The addition of copper into the structure of LDH allows to play with its oxidation state and oxide species. For example, a Cu‐Cu_2_O@CuMgAl‐LDH composite was synthetized from direct potentiodynamic electrodeposition over a carbonaceous conductive support of a CuMgAl‐LDH by Serafini et al.^[37]^ This synthetic method allows the creation of an intimate contact between Cu‐Cu_2_O species and the ternary LDH, with a ratio between Cu^0^ and Cu oxidized species of 1 : 6. The as‐prepared electrocatalyst displays very specific selectivity and activity towards electrocatalytic CO_2_‐to‐acetate conversion, showing a productivity up to 2 mmol h^−1^ g_cat_^−1^ of acetate with a faradaic efficiency (FE) of 84 % at – 0.4 V *vs* RHE in KHCO_3_ liquid phase.

CuMgAl‐LDH was studied as electrocatalyst for electrochemical CO_2_RR also by Lee and coworkers,^[294]^ which evaluated the effect of copper abundance in the pristine material on the structural evolution of the catalyst (in particular on the formation of reduced copper species) during the electroreduction reaction thus on the products formation.

Furthermore, LDH matrices can be transformed into active electrocatalytic species via electrochemical treatments or specific chemical conversions. Altaf and co‐workers produced Cu−Cu_2_O@LDH nanocomposites by electroreducing CuAl‐LDH or CuAl‐LDH@GO (with Cu/Al=5) at high cathodic potentials (−4 V or −2 V vs. Ag/AgCl for 2–4 hours).[^136^, ^295^] This process generated Cu_2_O nanoclusters dispersed on LDH layers. The optimized composites exhibited high hydrocarbon selectivity, achieving FEs for C_2_H_4_ of 40 % with ER‐CuAl‐LDH and 54 % with ER‐CuAl‐LDH@GO (−1.2 V vs. RHE). ER‐CuAl‐LDH/rGO demonstrated higher current density and capacitance, indicating a larger electroactive surface area and increased active sites for CO_2_RR, with conductive GO aiding in dispersing active Cu species and enhancing catalytic performance. Zhang et al. employed alkali etching to partially leach Al^3+^ from CuAl‐LDH (Cu/Al=4) before applying a cathodic conversion (−1.06 V vs. RHE), producing a CuNPs/AlOx catalyst with CO_2_ER efficiencies of 50 % for C_2_H_4_, 15 % for C_2_H_5_OH, 6 % for n‐C_3_H_7_OH, and 1 % for CH_3_COO^‐^.^[296]^

A Cu/Fe_3_O_4_ catalyst was produced by thermal reduction of CuFe‐LDH, with Cu atoms evenly distributed in the Fe_3_O_4_ matrix, achieving 51 % FE for ethanol at − 0.3 V vs. RHE.^[297]^ Further, CuZnAl‐LDH was used as a precursor for a Cu_1‐x_Zn_x_O/Cu_1‐γ_Zn_γ_Al_2_O_3_ composite, creating mixed oxides through calcination. Hence, under CO_2_RR conditions in KOH, CuZn oxide reduced to form a CuZn alloy while CuAl aluminate remained stable, achieving high FE for C_2_ products, including 36 % for C_2_H_4_, 36 % for C_2_H_5_OH, 13 % for CH_3_COO^‐^, and 3 % for C_3_H_7_OH.^[298]^

In general, the addition of LDHs to copper‐based electrodes demonstrates increasing the availability of CO_2_ and OH^−^ in proximity of catalytic active copper sites. Additionally, the use of Cu‐based LDHs as electrocatalysts is a widely applied strategy to obtain different active catalysts thanks to the formation of active copper species in different redox state achieved using electrochemical or chemical pretreatments.

## 5 Computational Insights into CO_2_ Activation on LDHs

As mentioned before, LDHs are structurally and electronically complex materials, with dynamical interlayer regions and a wide variability in their cationic and anionic compositions. In **Section** **4** we discussed how experimental composition changes affect CO_2_ activation and the outcomes of both photocatalytic and electrocatalytic processes. However, rationalizing how the composition change influences the electronic and surface properties, as well as the role of the interlayer region, remains a significant experimental challenge.

To complement experiments with further fundamental insights on key factors governing the properties of LDHs, computational techniques have been largely adopted.[^49^, ^52^, ^53^, ^239^, ^299^, ^300^, ^301^, ^302^, ^303^]

This section is organized as follows: first, the early computational studies regarding LDHs and employing MD simulations are outlined; then, more recent and advanced studies based on DFT calculations are reviewed. The former studies mainly focus on disclosing information regarding the structure and phenomena in the interlayer regions, while the latter works address mechanistic aspects of the photocatalytic and electrocatalytic activation of CO_2_.

### 5.1 Early Computational Challenges and Developments

Early computational studies of LDHs date back to the 1990s, driven by the growing interest in the technological applications of these compounds[^304^, ^305^] and the increasing recognition of computational methods as valuable tools in materials science.[^301^, ^306^]

Even if the development of experimental techniques such as powder XRD, FTIR, and near‐edge EXAFS (NEXAFS) spectroscopy already provided significant insights into the structure of LDH layers, the dynamic and disordered nature of interlayers was poorly understood and still an open question.^[57]^ Therefore, the computational research focused on having an accurate modeling of the interlayer region and, thus, swelling properties of LDHs.

Early structural models were usually based on classical MD simulations of the brucite (Mg(OH)_2_) or the naturally occurring hydrotalcite (MgAl‐CO_3_‐LDH).[^304^, ^307^] Newman et al.^[307]^ studied terephthalate intercalation into MgAl‐LDHs, employing a modified version of the Dreiding FF.^[308]^ Namely, Aicken et al.^[304]^ modified this FF by incorporating customized parameters for Mg and water molecules, the latest derived from the TIP3P water model.^[309]^ The inclusion of water in the model and its parametrization provided crucial insights, showing that water plays a pivotal role in the interlayer region, directly impacting the swelling properties. Newman et al. were, thus, able to show that the orientation of terephthalate anions in the interlayers strongly depend on the layer charges and water contents, in agreement with experimental data.

Contemporary to these studies, parallel efforts were done to parametrize FFs for LDHs.[^239^, ^310^] In particular Cygan et al. proposed a general FF for molecular simulations of clays, ClayFF, based on an ionic description of the metal‐oxygen interactions associated with hydrated phases.^[239]^ Interestingly, the authors based the parameterization of the FF on available experimental crystal structures rather than relying on quantum‐mechanical calculations alone. Thanks to this approach, MD simulations with ClayFF provided a good agreement between simulated and experimental FTIR spectra of LiAl_2_(OH)_6_Cl ⋅ H_2_O. Even today, ClayFF remains widely utilized in computational modeling of clay minerals and layered hydrated materials, demonstrating strong transferability and reliable predictive performance.^[311]^

### 5.2 Applying DFT to LDH Materials

Despite the significant insights obtained by classical MD simulations on LDHs, such studies could not provide information into the electronic structures of these materials, which are instead crucial for a deep understanding of interlayers interactions and molecules’ activation on the surfaces or in the interlayer region. The impressive advancements in the computational power and the developed theoretical frameworks in the last two decades enabled researchers to apply DFT for studying LDH‐based materials.[^301^, ^312^]

Apart from few examples using the cluster approach,[^313^, ^314^] slab supercells combined with plane‐wave DFT have been the most popular choice. In particular, GGA functionals have been the most used functionals for LDHs, with a special mention for PBE, which is the mostly employed along with corrections for dispersion effects and DFT+U refinements often included.[^49^, ^315^, ^316^]

In a pioneering study of 2002, Trave et al. used the PBE functional to carry out AIMD simulations to explore the non‐trivial relationship between the chemical composition and electronic properties of MgAl‐ and MgGa‐LDHs intercalated with Cl^−^ and OH^−^ anions.^[299]^ Fundamental characterization of the electronic structures and relevant insights on the contribution of the layers and interlayers of these systems were achieved. Namely, this work highlighted the significant influence of intercalated anions on the LDHs’ electronic properties, particularly on the top of the valence band (VBM) and the bottom of conduction band (CBM), which determine the basicity and are critical for the catalytic activity. Overall, this study revealed the existence of a strong host‐guest interaction.

Further advancements in the study of the interlayer region were achieved with the introduction of dispersion‐corrected DFT methods (already described in **Paragraph 3.2.3**). These corrections allow for a more complete description of interlayer species interactions and to clarify the contributions of different interaction terms. For instance, Hou et al. analysed the interlayers region of hydrated LiAl−A‐LDHs (A= F^−^, Cl^−^, Br^−^, OH^−^, NO_3_^−^, CO_3_^2−^, SO_4_^2−^) using PBE with DFT−D.^[317]^ By employing the reduced electron density gradient analysis, computations showed that hydrogen bonding, van der Waals forces, and electrostatic interactions govern the interlayers stability. Additionally, PDOS analysis revealed that oxygen states of oxygen‐containing anions (i. e. O 2p) and halogen (i. e. X 2p) contribute significantly to the CBM, meaning that they give a relevant contribution to the basicity. Al 3p states dominate instead the top of VBM. Overall, these results highlighted again strong host‐guest interactions.

By applying PBE and Tkachenko‐Scheffler vdW corrections to study MgAl−A‐LDHs with nine different anions (A= F^−^, Cl^−^, Br^−^, I^−^, OH^−^, NO_3_^−^, CO_3_^2−^, SO_4_^2−^,PO_4_^3−^), Liu et al. suggested that the electrostatic interactions is the dominating energetic term between layers and interlayers anion.^[302]^ The authors obtained similar results to Hou et al. for the contribution of anions to CBM and VBM. Zhao et al. explored properties of a bigger ensemble (i. e. 159) of M_n_Al−A‐LDHs (varying the M^2+^ cation identity, the n number, and the X anion), using AIMD.^[318]^ Also in this work, authors draw the conclusion that electrostatic interaction plays a major role in governing the interactions between layers and anions, allowing for rationalizing the experimental trends regarding the anion exchange ability of different LDHs. The authors observed that the critical factor of the exchange is the binding energy, which is dominated by electrostatics and correlates with the anion electronegativity, even if the contribution of hydrogen bonding cannot be neglected. Zhao et al. showed also that the nature of cations and the composition of the layers has not a relevant influence on the exchange phenomena.

Beyond the interlayer region, DFT‐based studies gave insights also into layer structures. Here, the key challenge is understanding the effects due to the variability in the M^2+^/M’^3+^ ratios and layer distributions. Remarkably, Yan et al. exploited DFT for studying MgAl−Cl‐LDHs varying stacking sequences, cation ratios, and distributions.^[319]^ The authors demonstrated that pure LDH phase is not stable for M^2+^/M’^3+^ ratios greater than 4. Moreover, ordered distributions of cations are favoured when the M^2+/^M’^3+^ ratios are low, i. e. 2–3. However, disordered structures become more stable for higher ratios since they avoid the formation of destabilizing Al−O−Al linkages, being, thus, preferred. Furthermore, the 3R polytype was identified as the most stable lattice configuration across all scenarios. Their work also showed that the cation ratio strongly influences the band gap of the material. In particular, by increasing the ratio the lattice gets more stabilized, and the band gap widens. This trend was more recently confirmed by other DFT studies.[^317^, ^320^]

Overall, DFT has significantly advanced the understanding of LDH systems by providing detailed insights into their electronic structures and interlayer interactions. These studies have clarified that: (i) electrostatic term governs the interaction between layers and interlayer species; (ii) anions have an impact on VBM and CBM, proving strong host‐guest interactions that can influence material reactivity, as anions could be the most basic sites; (iii) the cationic ratios and composition impact on the band gap and semiconducting properties.

### 5.3 Insights Into CO_2_ Activation

As discussed in **Paragraph 4.1**, LDHs exhibit a remarkable interaction with CO_2_, and have been investigated as promising materials for CO_2_ capture. The diffusion of CO_2_ into the LDH interlayer and its interaction with the surrounding species were modeled with classical FFs.^[321]^ Here, classical MD simulations have been successfully employed to predict sorption isotherms and effective diffusivity of CO_2_, providing quantitative agreement with experimental data for isotherms at low loadings and diffusivities at high temperatures. Larger discrepancies at high CO_2_ loadings and low temperatures were presumably attributed to the polycrystallinity of the LDH material, which modeling would require more significant efforts. Despite the relevant atomistic information obtained with this classical FFs study, the CO_2_ activation and CO_2_RR mechanistic studies inevitably require QM investigations. Zhang et al. studied the differences in CO_2_ activation between bulk and monolayer materials of ZnAl‐NO_3_‐LDHs.^[322]^ Remarkably, the study comprises a synergistic experimental‐computational approach, employing DFT computations and simulations of the PDOS of the materials. In absence of adsorbed CO_2_, the authors observed that the d‐band center of the bulk was closer to the Fermi level as compared to the monolayer one, which means a stronger activation on the bulk material.^[323]^ This outcome was corroborated by the computed adsorption energy of CO_2_ on the Al octahedron site in the bulk (−1.01 eV), signifying a strong interaction. Upon analysing the PDOS, the authors identified significant changes when CO_2_ was adsorbed on the bulk material, whereas minimal variations were observed between bulk and monolayer. This finding led to the hypothesis that CO_2_ is chemisorbed on the bulk material but only physiosorbed on the monolayer, in agreement with experimental trends of temperature‐dependent adsorption capacity of the LDHs. Notably, also Ai et al. exploited a combined experimental and computational approach to investigate CO_2_ adsorption on MgAl‐LDH modified with amino‐group species, i. e. (3‐aminopropyl)triethoxysilane.^[53]^ Their findings revealed that CO_2_ adsorption energy and diffusion are significantly influenced by the presence of water molecules. Consistently, their experiments showed a 56.8 % improvement in adsorption capacity following a 5‐hour drying process.

Computational studies focusing on the electrocatalytic and photocatalytic properties of LDHs have emerged only very recently.[^324^, ^325^, ^326^, ^327^] Regarding the CO_2_ activation, almost all computational efforts focused on the photocatalytic process,[^49^, ^52^, ^303^, ^316^, ^328^] and few studies on the electrocatalysis.[^34^, ^48^] All studies primarily characterized the electronic properties of the LDH material and then explored possible CO_2_RR paths, but computing only the reaction thermodynamics.

Notably, Song et al. used a synergistic experimental‐computational strategy to investigate a composite photocatalyst, NiAl‐LDH, with Ru(bpy)_3_Cl_2_ as the photosensitizer, with variable number of layers.^[49]^ The materials were modeled with the slab supercell approach and considering the (003) facet, with eight models considered by varying the surface defects and the layers configuration (i. e., monolayer, m‐NiAl‐CO_3_‐LDH; multilayer b‐NiAl‐CO_3_‐LDH). The plane‐wave DFT+U method, with Hubbard correction for Ni, was used to compute the free energy profiles for CO_2_ reduction to CO and CH_4_. Proton‐coupled electron transfer (PCET) steps were modeled using computational standard hydrogen electrode (CHE).^[329]^ The study revealed that the potential energy barriers for the CO_2_‐to‐CH_4_ reduction were significantly lowered in the presence of both Ni and OH defects on the surface of the thick‐material m‐NiAl‐CO_3_‐LDH (from 0.329 eV to 0.163 eV), emphasizing the critical role of surface defects in CO_2_ activation. The authors also performed Time‐Dependent DFT (TDDFT) calculations of the Ru(bpy)_3_Cl_2_ complex, demonstrating favourable alignment with the energy levels of the m‐NiAl‐LDH. This alignment determines a driving force that is enough for reducing CO_2_ to CH_4_, but does not allow the hydrogen evolution reaction, in agreement experimental observations. Overall, this study highlighted the relevance of synergistic computational‐experimental studies.

**Figure 11:**
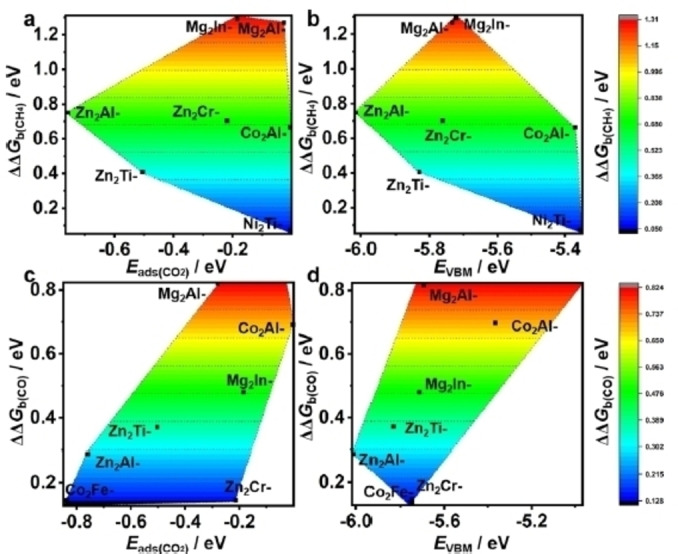
Relationships computed by Zhao et al. for different MM′‐NO_3_‐LDHs. (a) The relationship between the effective driving force reduction of CO_2_ (ΔΔG_b_) to (a) CH_4_ or (c) CO and adsorption energy of CO_2_, or (b,d) VBM energy. Reprinted with permission from reference [52]. Copyright 2022, American Chemical Society

Zhao et al., using a similar computational set up as Song et al., performed a broader computational study investigating the electronic structure and thermodynamic mechanisms of CO_2_ photoconversion across MM′‐NO_3_‐LDHs with various cation compositions (M^2+^=Mg^2+^, Co^2+^, Ni^2+^, Zn^2+^; M’^3+^=Al^3+^, Cr^3+^, In^3+^, Fe^3+^, Ti^4+^).^[52]^ They also focused on the (003) facet, systematically characterizing the materials by computing the electronic properties, band structures, and DOS. The authors observed that for a fixed M′^3+^ cation, increasing the number of d‐electrons of M^2+^ resulted in a decreased band gap, enhancing sensitivity to visible light. The presence of transition metals significantly influences the VBM and CBM, thus impacting photocatalytic performance. To study the CO_2_PR process, the authors quantified the reduction reaction driving force, which defines the thermodynamic feasibility of CO_2_PR. Also, the effective driving force (ΔΔG_β_), i. e. the difference between the CO_2_PR driving force and the Gibbs thermodynamic barrier of the most favourable pathway, was computed. Correlations between computed ΔΔG_β_, CO_2_ adsorption energies, and VBM levels were found, as shown in Figure 11. In particular, among the investigated systems, the Mg_2_Al‐NO_3_‐LDH featured the largest driving force and CO_2_ adsorption energy, in line with the favored CO production that was experimentally found by Teramura et al.^[30]^ These findings underscore the pivotal role of strong CO_2_ activation in achieving high photocatalytic performance and show how broad/screening studies are crucial for determining structure–properties correlations

While Zhao et al. used only ground state methods for exploring the reaction mechanisms, an important step further towards more sophisticated approaches was taken very recently by Wang et al.^[303]^ These authors investigated the photo‐induced electronic structure and photocatalytic mechanisms of CO_2_PR over ZnAl−Cl‐LDH under both UV and deep‐UV (DUV) excitations, using constrained DFT (CDFT)^[330]^ and TDDFT calculations for computing excited state properties. First, the authors chose a cluster approach, using a minimal size model, i. e. [Zn_4_Al_2_(OH)_12_(OH_2_)_10_] ⋅ 2Cl, with capping H atoms, to study the basic photophysical properties of the system. Although such small size model could lack in describing the confined‐space effect of the interlayer species, the reduced size allowed performing standard TDDFT computations on a molecular species. The authors observed that the main UV excitation has a charge transfer (CT) character with long‐range electron‐hole separation. Holes are primarily contributed by Cl atoms, while Zn, Al, and O contribute to electrons. The computation of parameters like the distance between the centroids of electron and hole density, the spatial overlap of electron and hole densities, and an index for describing the separation of electron‐hole densities, quantitatively suggested a good electron‐hole separation under UV excitation in the ZnAl−Cl‐LDH system. On the contrary, the deep‐UV excitation has a localized character, with a significant overlap between electrons and holes. To investigate the CO_2_PR process, the authors instead applied a periodic slab supercell model, while using CDFT. This approach included excited‐state effects into the reaction mechanism by simulating electron promotion. By computing thermodynamics, the authors were able to show that the required potential associated to the rate determining step for CO formation (HCCO* à CO*, where * indicates surface species) significantly decreases from 1.02 eV (ground state) to 0.67 eV (excited state) due to the charge delocalization, highlighting the importance of including excited states computations for the characterization of the CO_2_PR processes.

### 5.4 Role of Cu Into CO_2_ Activation

In recent years, Cu was shown to be a pivotal element in CO_2_ photo‐ and electro‐reduction since it allows to selectively obtain CH_3_OH or C_2_ products, as discussed in **Section** **4**. However, only a few computational studies have been focused on Cu‐based LDHs and Cu/LDHs.

Regarding LDHs used for CO_2_PR, Xu et al. exploited DFT+U to elucidate the role of Cu doping for activation of CO_2_ on ZnAl‐LDH.^[328]^ The authors investigated the effects of Zn or O vacancies and Cu doping on the structure, the stability, and the catalytic activity of LDHs catalyst by means of slab supercell models. Atomic defects and Cu doping were found to localize charge density on the surface, especially around unsaturated Znδ^+^/Cuδ^+^ sites, which can act as reactive centers, as also confirmed by computed electron localized function. The introduction of defects and Cu atoms was found to create an intermediate band constituted by Zn‐4 s and Cu‐3d orbitals. The d‐band center moves closer to the Fermi level with respect to the undoped material and therefore a better activation of the CO_2_ can take place (in agreement with d‐band center theory), showing that Znδ^+^/Cuδ^+^ are good reaction sites. Consistently, the thermodynamic barrier toward CO formation was predicted to decrease in the Cu doped and Zn‐defective LDH by 0.33 eV with respect to pure ZnAl‐LDH. It is also worth noticing that the appearance of the intermediate band reduces the bandgap and potentially enhances electron‐hole separation and carrier migration, meaning a greater photocatalytic efficiency for the doped material.

**Figure 12:**
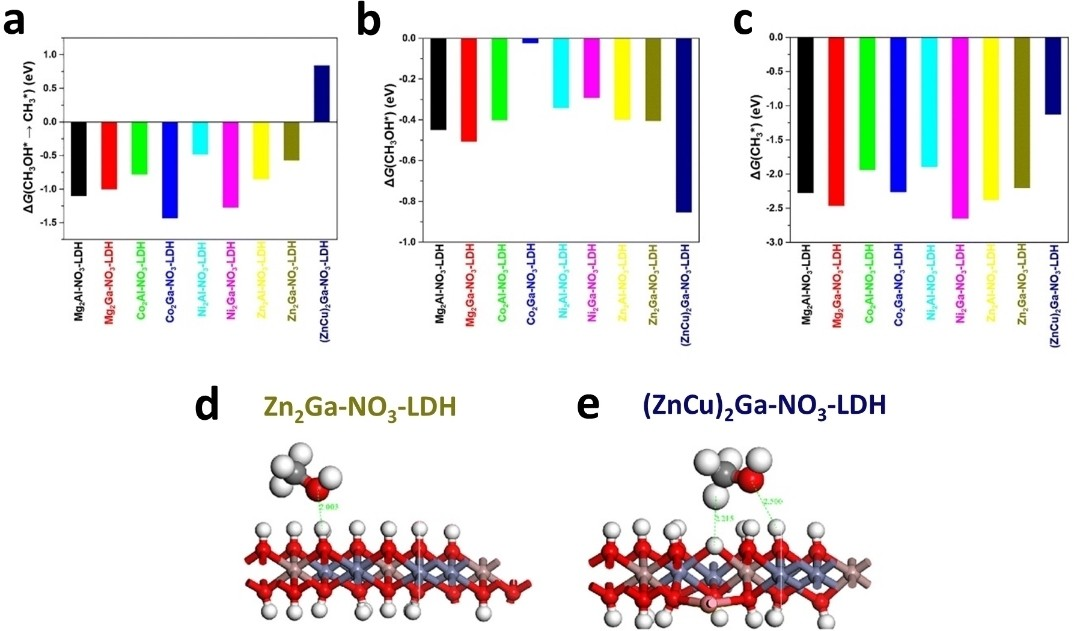
Gibbs free energy changes for (a) CH3OH* conversion to CH3*, (b) adsorption of CH_3_OH and (c) CH_3_ on different M_2_M’‐NO_3_‐LDH. The geometries of adsorbed CH_3_OH for (d) Zn_2_Ga‐NO_3_‐LDH and (e) (ZnCu)_2_Ga‐NO_3_‐LDH are reported. Adapted from reference [316] with permission from The Royal Society of Chemistry; permission conveyed through Copyright Clearance Center, Inc.

Zong and co‐workers investigated the effect of Cu doping on Zn_2_Ga‐LDH, also using DFT+U, comparing it with other M_2_M’‐NO_3_‐LDH (M^2+^=Mg^2+^, Co^2+^, Ni^2+^, Zn; M′^3+^=Al^3+^, Ga^3+^) undoped materials.^[316]^ For pure LDH systems, the standard electronic properties and driving forces were computed, showing that these materials could theoretically reduce CO_2_ to common photoproducts, such as CO, CH_4_, HCOOH, and CH_3_OH. This is because their CBM lie above −0.61 V vs. SHE, which is the standard potential for HCOOH formation from CO_2_ reduction (the highest between all the products). Interestingly, in absence of Cu sites, the CH_3_OH* surface species could either desorb or be reduced to CH_3_* with favourable energetics. The introduction of Cu on Zn_2_Ga‐LDH (i. e. (ZnCu)_2_Ga‐LDH) was predicted to significantly alter the reaction energetics. Namely, the authors found interesting trends for the free energies of CH_3_OH* dehydration to CH_3_*, i. e. ΔG(CH_3_OH* ‐> CH_3_*), and the adsorption energies of CH_3_OH*, i. e. ΔG(CH_3_OH* ), and CH3*, i. e. ΔG(CH_3_*), for all the investigated LDHs, as reported in Figure 12. While the CH_3_OH reduction process was found to be exergonic (by ca. −0.50 eV) for Zn_2_Ga‐LDH, it was shown to be endergonic (by ca. 0.84 eV) for the Cu‐doped material. This shift was attributed to the stronger adsorption energy of CH_3_OH (ca. −0.9 vs. −0.4 eV) and the weaker one of CH_3_ (−1.1 vs. −2.2 eV) on Cu‐doped LDHs with respect to pure LDH materials, respectively (Figure 12). Structural analysis revealed that the presence of Cu altered the adsorption mode of CH_3_OH, allowing it to form an extra hydrogen bond with the surface near the Cu site (Figure 12). This behaviour was rationalized in terms of the Jahn‐Teller effect associated with Cu, which modifies its coordination. Namely, octahedral hexa‐coordination, typical of most metal cations in LDHs, is less suitable for Cu atoms due to the Jahn‐Teller effect, as also previously observed.^[331]^ These structural modifications induced by Cu‐doping, thus, can effectively tailor the LDH to selectively produce CH_3_OH.

**Figure 13:**
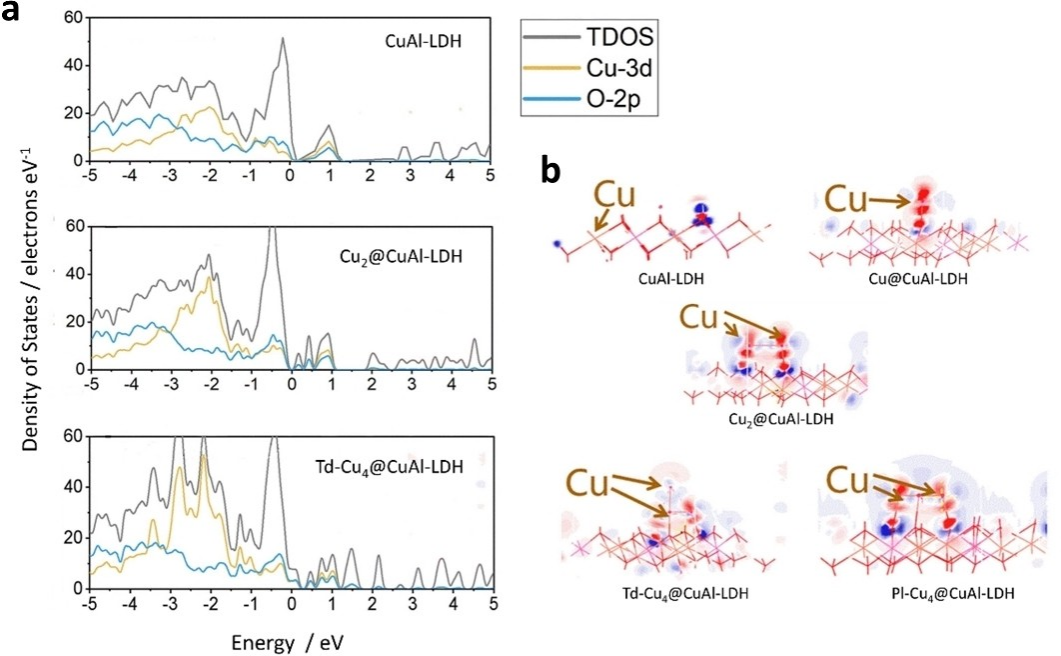
(a) Total density of states (TDOS) and partial density of states (PDOS) of CuAl‐LDH, Cu_2_@CuAl‐LDH and Td−Cu4@CuAl‐LDH. (b) Differential charge density map of the investigated LDHs; red represents depletion of charge, while blue represents the accumulation of charge. Adapted from reference [48] with permission from The Royal Society of Chemistry; permission conveyed through Copyright Clearance Center, Inc.

While several computational studies on the LDHs for CO_2_PR were reported, there is, to the best of our knowledge, only one study focusing on the electrocatalytic activation of CO_2_ on Cu/LDHs.^[48]^ In this case the role of Cu species adsorbed on the LDH surface was found to be quite interesting, since it can allow the formation of C_2_ product. Xu et al. examined five CuAl−Cl‐LDHs featuring Cu surface decoration with various configurations: monoatomic, diatomic, orthotetrahedral (Td) Cu_4_ cluster, and planar (Pl) Cu_4_ cluster, using DFT+U.^[48]^ As observed in previous computational studies on Cu/LDHs,^[328]^ Cu was found to be involved in the formation of broader peaks near to the Fermi energy level (see total DOS (TDOS) and PDOS in Figure 13a), indicating promising semiconductor properties for Cu/LDHs materials. The differential charge density mapping revealed that Cu atoms on the surface of CuAl−Cl‐LDHs are oxidized and feature positive charges (Figure 13b). Using these structural models, the authors have investigated the reaction mechanisms of the electrocatalytic activation of CO_2_ toward CH_3_CH_2_OH and C_2_H_4_. The thermodynamics of the processes were computed, and the effect of the electrode potential was considered only for the electrochemical steps involving PCET, by means of the CHE approach. The authors observed various distortions of the O−C‐O bond angle at the Cu active sites of the decorated catalysts, suggesting a chemisorbed CO_2_ surface species and in contrast with the physisorbed CO_2_ on the CuAl−Cl‐LDH surface. In particular, the positively charged top site of the Cu_4_ cluster on Td‐Cu_4_@CuAlCl‐LDH showed to be the preferential adsorption site for CO_2_. Notably, from the electrocatalytic point of view, this catalyst exhibited the most favourable reaction energy profiles for C−C coupling, featuring the lowest energy barrier (0.68 eV) for the chemical C−C coupling step, as well as the lowest reaction energy (0.78 eV), with an overpotential of 0.71 V (being in the range of 1–2 V for other catalysts). This outcome highlighted the role of positively charged Cu sites on the LDHs surface and the influence of Cu active structures, which can operate at a low applied voltage to form C_2_H_4_ from CO_2_.

## 6 Conclusions and Outlooks

We have reviewed both experimental and computational studies on the activation and conversion of CO_2_ on LDHs through photocatalysis and electrocatalysis, with particular emphasis on the role of Cu. Our aim is to provide a starting point that offers essential background ‐ both theoretical for experimentalists and experimental for theoreticians ‐ to support the development of tailored LDH materials for cost‐effective and sustainable CO_2_ valorisation.

On the experimental side, LDHs proved to be promising materials for carbon capture and utilization through advanced and sustainable catalytic processes, such as photocatalysis and electrocatalysis. Several experimental studies have extensively investigated the Cu‐based LDHs, demonstrating the effectiveness of Cu species in promoting CO_2_ activation and conversion toward valuable chemicals such as CO, CH_4_, C_2_H_4_, CH_3_OH, HCOO^−^, and CH_3_COO^−^, recognizing to LDHs a fundamental structural role of substrates with appropriate active sites and promotors of CO_2_ adsorption thanks to the surface basicity typical of hydroxides layers. Still, many open questions regarding CO_2_ activation and reaction mechanisms occurring over LDHs remain, especially when active phases based on Cu species are enclosed in the hydroxide layered structures. *In situ* and *operando* techniques are recognized as essential to find answers for these questions that are critical for unlocking the full potential of Cu/LDHs in catalytic applications and to allow large scale production of C_2+_ high value‐added chemicals from CO_2_.

Notably, while the studies reviewed here have extensively investigated Cu‐based LDH photocatalysis and electrocatalysis, their extended application for photoelectrocatalytic CO_2_ conversion remains underexplored. A recent notable attempt by Liu et al., investigated Cu‐based LDH for CO_2_ conversion in a photo‐assisted aqueous Zn–CO_2_ battery device.^[332]^ The proposed Cu_2_O/CuCoCr‐LDH photocathode, fabricated through in situ reduction of a CuCoCr‐LDH precursor, achieved superior photoelectrocatalytic conversion of CO_2_ (to mainly CO) with respect to electrocatalysis in dark conditions, demonstrating the high potential of LDHs materials for photoelectrochemical CO_2_RR applications. This route represents an appealing option to reduce the external energy required by conventional electroreduction processes. Its coupling with cutting‐edge technologies, such as metal‐CO_2_ batteries, could push forward further applications of LDHs materials, also in more traditional photoelectrolysis systems.

On the computational side, few studies on CO_2_ photocatalytic conversion on LDHs have been published and only in recent years, reflecting an emerging interest in the topic, with even fewer studies focusing on electrocatalytic conversion. Nonetheless, computational investigations have provided so far valuable insights into the role of Cu in both cases. DFT simulations showed how Cu‐doping impact the LDHs electronic structures by introducing a d‐band closer to the Fermi level compared to pure materials, enhancing CO_2_ activation. Additionally, when incorporated into the hydroxide layer, Cu can induce local surface distortions through the Jahn‐Teller effect. These distortions improve the interactions with methanol molecules, providing a possible explanation for its preference over CO as a product in photocatalytic CO_2_ reduction.

Besides these advancements, most computational studies primarily focused on the electronic properties of LDHs, with mechanistic studies remaining largely limited just to prediction of surface reactions thermodynamics. Kinetic aspects have been so far ignored in these computational studies, mainly due to the challenges of modeling transition states (TS), particularly for PCET processes, in both photocatalysis and electrocatalysis. Moreover, photocatalytic mechanisms are still predominantly studied in the ground state, neglecting the role of excited states, with recent advances in computational methods promising further developments in this field..[^260^, ^333^] One recent example^[303]^ has demonstrated that reaction energies can differ significantly when excited‐state chemistry is considered for studying CO_2_PR catalyzed by LDHs, in agreement with a recent work on Cu/TiO_2_.^[334]^ This could be the case also for electrocatalysis, where methodologies more sophisticated than CHE (like grand canonical DFT),^[335]^ successfully used for other layered semiconductor materials,^[336]^ could provide a description of the effect of the electrode potential and electrical double layer on the adsorption and activation of CO_2_.

We showed how synergistic experimental‐computational studies efforts proved to be effective in providing a more holistic understanding of CO_2_ activation on LDHs, especially underlying differences between bulk and monolayer materials. This integrated approach has definitively the potential to elucidate complex reaction mechanisms and refine the design of LDH‐based catalysts. In our opinion, future efforts should prioritize such synergistic efforts to advance this research field, directing toward elucidating the following aspects:

- Interlayer Chemistry: The interlayer region of LDHs, inherently dynamic in nature, may host active sites and play a significant role during reactions. So far this has been poorly investigated from both computational and experimental points of view. On the experimental hand, the possibility of exchanging gaseous carbon dioxide with interlayered carbonates has been demonstrated. However, in the complex interlayer environment, which can be seen as a nanoreactor, inner metal active sites, CO_2_ and anions might present differences in electronic configuration with respect to their analogues on the outer surface of the LDH particle, leading to an alteration of free energies at stake. A synergistic experimental‐computational approach might help in disclosing possible variations of physico‐chemical properties of interlayer species helping a deeper understanding of reactivity and conversion pathways. In‐operando spectroscopic techniques (Raman, FTIR, XRD, XAS) represent powerful tools for this purpose. Meanwhile, recent advancements in machine learning‐based FFs (MLFF) have enabled MD simulations to characterize reactivity in heterogeneous systems with dynamically evolving active sites.[^337^, ^338^] This computational approach holds potential for extension to the study of CO_2_ conversion within dynamic LDH interlayers.
- Identification of active copper species for the different reactions: The possibility to insert Cu in the cationic layer of LDH or in the interlayer as anionic complex, together with the use of pretreatments allows to tune the copper oxidation states and the interaction between copper and the other components of the material. However, further insights must be gained to disclose the effect of each copper species contributing to the formation of specific intermediates. This should be addressed experimentally by thoroughly characterizing the final catalyst (i. e. the system actually working in the reaction conditions) for instance by in situ techniques and supporting these findings with computational modeling.
- Machine Learning predictions: Identifying structure‐property relationships for CO_2_ conversion remains a critical challenge. While computational studies have identified, so far, relevant correlations, such as that between CO_2_ adsorption energy and in CO_2_RR driving force or the VBM position and CO_2_ reduction yield,^[52]^ a more comprehensive understanding is needed. The development of large databases and artificial neural networks predictive models can accelerate the discovery of optimized materials, like in the case of other catalytic systems.^[339]^ The correlation of these database with experimental data would further push this innovative field, while advancements in synthetic methods, such automatization, can enable scientists to experimentally produce those new materials identified by computational predictions while keeping up with the speed of these in generating new discoveries.

To conclude, important results have been achieved up to date in the field of carbon dioxide photo‐ and electro‐ reduction employing Cu‐based LDHs as catalysts. These achievements have further pushed the conversion of carbon dioxide into valuable products, but also highlighted the complexity of the involved systems, where different components act synergistically throughout the reaction and where the nature of the final catalytic species and active site may unravel only during reaction. This intriguing complexity may stimulate scientist of different sectors and calls for a synergistic interdisciplinary approach in a field where the combination of experimental and computational approaches and their continuous dialogue is crucial to getting a comprehensive understanding of the subject.

## Authors Contributions

F.L. and E.T.B. carried out literature research and wrote the first draft. F.C. reviewed the draft. All the authors reviewed the final version of the manuscript and discussed the research papers to be discussed in this review. F.B. and I.R. supervised the work and provided financial support.

## Biographical Information

*Fabio Loprete is currently a PhD student in Industrial Chemistry at the University of Bologna, where he also obtained his MSc in Industrial Chemistry in 2023. His research interests focus on the computational simulation of hybrid organic‐inorganic interfaces, with a particular focus on thermo‐ and electro‐catalytic processes involving either small molecules or biomass‐derived compounds*.

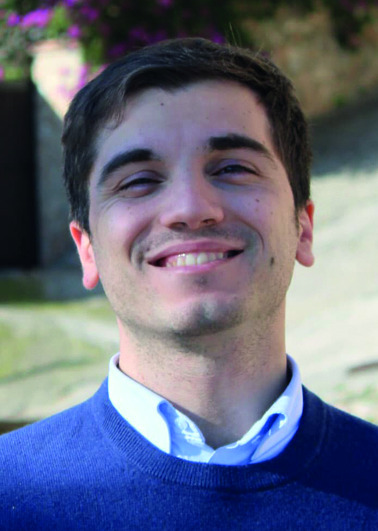

## Biographical Information

*Eleonora Tosi Brandi obtained her PhD in Industrial Chemistry at the University of Bologna in 2025, under the guidance of Prof. Francesco Basile, working on LDH‐catalysed photoelectrochemical CO_2_ conversion. She is currently a postdoctoral fellow at the same institution. Her research focuses on exploring LDH‐based catalysts for photochemical, electrochemical and photoelectrochemical CO_2_ conversion and water splitting*.

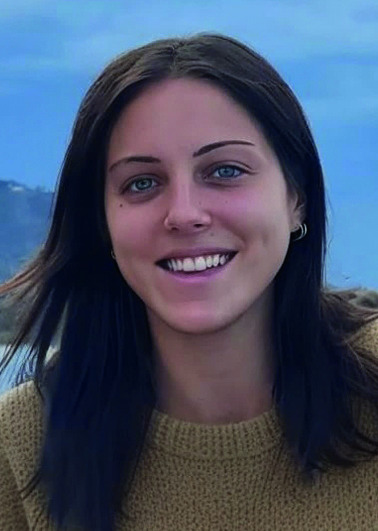

## Biographical Information

*Francesco Calcagno received the PhD in Chemistry at the University of Bologna in 2025, where he also obtained his MSc in Industrial Chemistry. His research interests focus on the application and development of computational quantum mechanics methods for homogeneous catalysis. His work ranges from reaction mechanism characterization to spectroscopy, artificial intelligence, and quantum computing*.

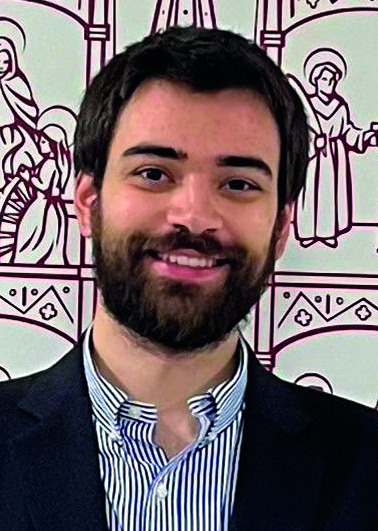

## Biographical Information

*Jacopo De Maron earned his PhD under the guidance of Prof. Fabrizio Cavani in 2020. After postdoctoral research at the University of Bologna in collaboration with chemical companies (2020‐2023), he joined the “Toso Montanari” Industrial Chemistry Department as assistant professor. His research interests span from the development of heterogenous catalysts for the valorization of waste and biomasses to the production of hydrogen and other energy vectors by thermochemical, electrochemical and photochemical processes*.

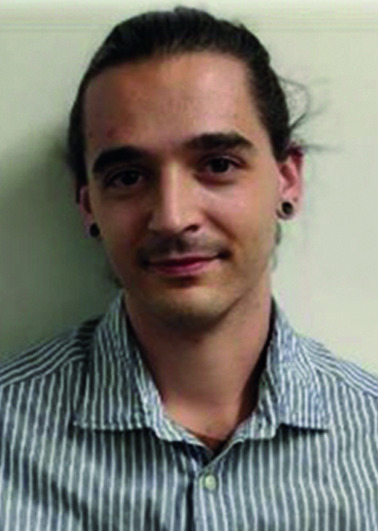

## Biographical Information

*Andrea Fasolini obtained is PhD in 2020 in Industrial Chemistry from the University of Bologna, with a project on the production of pure hydrogen from methane using membrane reactors. He continued his career as a Postdoc fellow starting new projects regarding CO_2_ utilization, electrocatalysis, photocatalysis, and photoelectrocatalysis with LDHs as well as in the field of high temperature hydrogen production and separation with Ce based materials. In 2022 he became Research Fellow at the Catalysis for Renewables and Innovative Processes Group at the University of Bologna*.

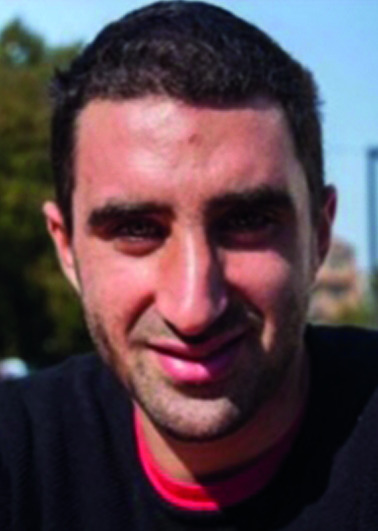

## Biographical Information

*Riccardo Tarroni was born in Alfonsine, Italy, in 1960. He received his PhD in Chemistry in 1990, after having carried out work in the field of physical chemistry of liquid crystals. He became an assistant professor at the University of Bologna in 1992 and an associate professor in 2005 at the same university. He has more than 100 publications with scientific interests covering topics from fluorescence depolarization in liquid crystals to computational spectroscopy and mechanisms of homogeneous catalysis*.

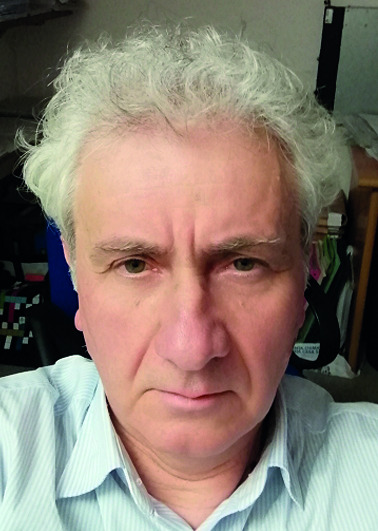

## Biographical Information

*Francesco Basile is Full professor at the Industrial Chemistry Department of the University of Bologna in Italy. He is working in catalysis and photo‐electrocatalysis with particular focus on hydrogen and fuel production form different sources and on hydrogen and CO_2_ conversion. He has published more than 100 papers in international journals and he is inventor and author of ten independent patent, a significant number of which on LDH derived catalysis. He is the Italian delegate in the Horizon Europe climate energy and mobility thematic configuration and in the hydrogen working group of the strategic energy technology plan (SET‐plan)*.

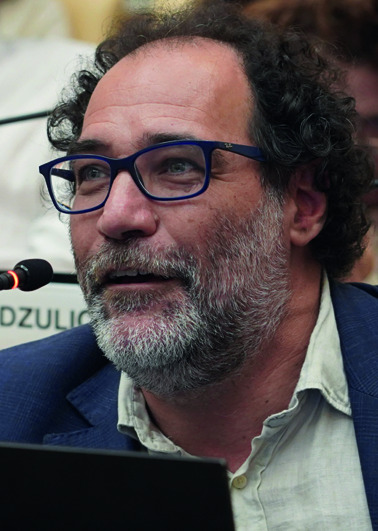

## Biographical Information

*Ivan Rivalta is Associate Professor at the Industrial Chemistry Department of the University of Bologna, Italy. He works in the field of theoretical and computational chemistry, with a focus on chemical and photo‐chemical phenomena involving molecular and macromolecular (biological and bio‐mimetic) systems and materials. The method developments and computer simulations aim at direct comparisons with experiments to obtain detailed interpretations of physico‐chemical and spectroscopic properties and to provide accurate predictions for in silico design of innovative molecules and materials. He is Director of the Bachelor's Degree in Materials Science of the University of Bologna*.